# Sugar metabolism reprogramming in a non-climacteric bud mutant of a climacteric plum fruit during development on the tree

**DOI:** 10.1093/jxb/erx391

**Published:** 2017-11-25

**Authors:** Macarena Farcuh, Bosheng Li, Rosa M Rivero, Lyudmila Shlizerman, Avi Sadka, Eduardo Blumwald

**Affiliations:** 1 Department of Plant Sciences, University of California, USA; 2 CEBAS, CSIC, Murcia, Spain; 3 Department of Fruit Tree Sciences, ARO, Israel

**Keywords:** Bud mutant, climacteric, fruit ripening, non-climacteric, plum, sugar metabolism reprogramming, systems biology

## Abstract

We investigated sugar metabolism in leaves and fruits of two Japanese plum (*Prunus salicina* Lindl.) cultivars, the climacteric Santa Rosa and its bud sport mutant the non-climacteric Sweet Miriam, during development on the tree. We previously characterized differences between the two cultivars. Here, we identified key sugar metabolic pathways. Pearson coefficient correlations of metabolomics and transcriptomic data and weighted gene co-expression network analysis (WGCNA) of RNA sequencing (RNA-Seq) data allowed the identification of 11 key sugar metabolism-associated genes: sucrose synthase, sucrose phosphate synthase, cytosolic invertase, vacuolar invertase, invertase inhibitor, α-galactosidase, β-galactosidase, galactokinase, trehalase, galactinol synthase, and raffinose synthase. These pathways were further assessed and validated through the biochemical characterization of the gene products and with metabolite analysis. Our results demonstrated the reprogramming of sugar metabolism in both leaves and fruits in the non-climacteric plum, which displayed a shift towards increased sorbitol synthesis. Climacteric and non-climacteric fruits showed differences in their UDP-galactose metabolism towards the production of galactose and raffinose, respectively. The higher content of galactinol, myo-inositol, raffinose, and trehalose in the non-climacteric fruits could improve the ability of the fruits to cope with the oxidative processes associated with fruit ripening. Overall, our results support a relationship between sugar metabolism, ethylene, and ripening behavior.

## Teaser

Using a systems biology approach, we identified and functionally assessed differences in sorbitol accumulation and of other major and minor sugars in Sweet Miriam, a non-climacteric bud mutant of the climacteric plum cultivar Santa Rosa.

## Introduction

Fruit ripening behavior has been classically defined as either climacteric or non-climacteric based on the existence or absence of a burst in respiration rate, respectively (Kidd and West, 1925). An increase of autocatalytic ethylene biosynthesis accompanies climacteric ripening (Biale and Young, 1981; Kidd and West, 1925). Climacteric fruits, such as tomato, banana, and most stone fruits, have the capacity for ripening if detached from the tree at the mature stage; while non-climacteric fruits, including strawberry, grape, and citrus fruits, cannot proceed to maturity post-harvest (Klee and Giovannoni, 2011; Grierson, 2013). Both ripening types can be found in the same species, as reported in melon (Obando-Ulloa *et al.*, 2008). Japanese plum (*Prunus salicina* L.) cultivars, categorized as climacteric, have been reported to differ in their ripening patterns (Abdi *et al.*, 1997, 1998; Zuzunaga *et al.*, 2001; El-Sharkawy *et al.*, 2007; Singh and Khan, 2010; Kim *et al.*, 2015*a*; Minas *et al.*, 2015). While the cultivar Santa Rosa presents a climacteric behavior (Zuzunaga *et al.*, 2001; Kim *et al.*, 2015*a*), its bud sport mutant, Sweet Miriam, displays a non-climacteric behavior (Kim *et al.*, 2015*a*; Minas *et al.*, 2015). The occurrence of both climacteric and non-climacteric types in fruits with the same genetic background offers an ideal experimental system for the study of the mechanisms controlling ethylene-dependent and ethylene-independent fruit maturation.

In stone-fruits, including Japanese plums, fruit growth follows a double-sigmoid pattern, defined by four distinct stages (Chalmers and Ende, 1975; Trainotti *et al.*, 2003; El-Sharkawy *et al.*, 2007; Lombardo *et al.*, 2011). The first exponential growth phase (S1) involves cell division and elongation; pit hardening (phase S2) is characterized by endocarp hardening and almost no increase in fruit size. In the second exponential growth phase (S3), cell division is resumed and the fruit reaches its final size. The fruit ripening stage (S4) is further divided into S4-I, where commercial harvest takes place, and S4-II, where the fruit reaches its full ripeness. Although Santa Rosa and Sweet Miriam were similar in fruit weight and size at the fully ripe stage, Sweet Miriam displayed longer S2, S3, and S4 stages and needed ~100 more days to reach S4-II as compared with Santa Rosa (Kim *et al.*, 2015*a*).

Sugars are synthesized in leaves and translocated to fruits (Yamaki, 1994). They provide energy and carbon structural sources during fruit growth (Hu *et al.*, 2016; Li *et al.*, 2016) and contribute to overall fruit taste, as their content and composition largely determine fruit sweetness (Borsani *et al.*, 2009; Dai *et al.*, 2016). In Japanese plums, as in other members of the *Rosaceae* family, the sugar-alcohol sorbitol (Sor) is translocated to the fruit along with sucrose (Suc) (Okie and Ramming, 1999). Sor synthesis is catalyzed by the enzyme sorbitol-6-phosphate-dehydrogenase (S6PDH) that mediates the reduction of glucose-6-phosphate (G6P) to sorbitol-6-phosphate (Teo *et al.*, 2006; Suzuki and Dandekar, 2014; Suzuki, 2015). Sor breakdown is mediated by the activities of NAD^+^-dependent sorbitol dehydrogenase (NAD^+^-SDH) and sorbitol oxidase (SOX), which catabolize Sor into fructose (Fru) and glucose (Glu), respectively (Bianco and Rieger, 2002; Teo *et al.*, 2006).

Previously, we reported that at the fully ripe stage, Sweet Miriam fruits displayed higher Sor contents, with significantly higher and lower specific activities of S6PDH and NAD^+^-SDH, respectively (Kim *et al.*, 2015*a*). Moreover, Sweet Miriam fruits showed lower Glu and Fru contents that were associated with increased Suc catabolism at the fruit S4-II stage (Kim *et al.*, 2015*a*). In addition to the major sugars Suc, Sor, Glu, and Fru, fruits also contain sugars that are present in significantly lower concentrations, including galactose (Gal), galactinol (Gol), raffinose (Raf), myo-inositol (Ino), and trehalose (Tre), among others. We hypothesized that differences in ripening behavior between the two cultivars could be associated with modifications in sugar metabolism and that the characterization of key sugar metabolic pathways could contribute to a better understanding of the changes operating in the bud sport mutant. Here, we used a systems biology approach to identify and characterize differences in Sor accumulation as well as changes in other major and minor sugars in fruits displaying contrasting ripening behaviors, a typical climacteric Japanese plum Santa Rosa and its non-climacteric bud sport mutant Sweet Miriam. We integrated gene expression profiles to identify key nodes in gene networks associated with the sugar metabolism reprogramming in the non-climacteric fruits. The expression patterns of these genes were further validated based on transcript levels, and the functions of gene products were assessed enzymatically and by metabolite analyses in fruits and leaves.

## Materials and methods

### Plant material

Throughout two seasons, fruits and leaves from Japanese plum (*Prunus salicina* L.) cultivars Santa Rosa and Sweet Miriam grafted on Myrobalan 29C (*P. cerasifera* Ehrh.) were harvested from a commercial orchard in Parlier, CA, USA. Trees were planted at a density of 450 trees ha^–1^. Six biological replications, of 20 fruits each, as well as a pool of at least 50% fully expanded leaves (the 5–6 most proximal to each harvested fruit) (Marchi *et al.*, 2005), were collected during the morning (08.00–10.00 h) at four developmental stages: S2 (pit hardening); S3/S4 (between the end of the second exponential growth phase and the onset of ripening; mature); S4-I (commercial harvest); and S4-II (fully ripe) (Table 1). The fruits were transported to the laboratory and analyzed the same day for ripening patterns and physicochemical properties, as described below. For each biological replication, six fruits were used for the evaluation of ripening patterns and physicochemical parameters, while the rest of the fruits were washed, peeled, cut into small pieces, pooled together, frozen in liquid nitrogen, and stored at −80 °C for further analyses. Leaves were processed likewise and stored at −80 °C.

**Table 1.** Early development and ripening-related stages in leaves and fruits of climacteric Santa Rosa and non-climacteric Sweet Miriam Japanese plum cultivars collected in this study

Developmental stage	Description/name	Fruit flesh firmness (N)	Fruit weight (g)		Harvest date (d)	
			Santa Rosa	Sweet Miriam	DAFB (Santa Rosa)	DAFB (Sweet Miriam)
S2	Pit hardening	70.1 ± 3.1	31.5 ± 4.2	29.5 ± 4.1	~95	~115
S3/S4	Mature	40.6 ± 0.7	82.9 ± 3.4	78.8 ± 5.1	~110	~152
S4-I	Commercial harvest	30.8 ± 0.8	84.6 ± 4.2	81.9 ± 4.5	~116	~199
S4-II	Fully ripe	19.6 ± 1.1	87.3 ± 4.8	89.2 ± 5.1	~123	~223

DAFB, days after full bloom.

### Fruit growth and development

Fruit growth and development patterns were monitored weekly [starting after natural fruit drop, ~80–85 days after full bloom (DAFB)] in both cultivars, including diameter, skin color, firmness, and fresh weight. To assess diameter, three biological replicates, each consisting of 10 fruits on one independent tree, were tagged and measured, as described before (Kim *et al.*, 2015*a*). For the rest of the parameters, five biological replicates of three fruits each, taken from independent trees, were evaluated (Kim *et al.*, 2015*a*). Using these data, but particularly fruit firmness as the maturity index (Crisosto, 1994) ranging between ~70 N and ~20 N from S2 to S4-II, respectively, samples of Santa Rosa and Sweet Miriam fruits and leaves were collected at four developmental stages (Table 1).

### Fruit ripening patterns and physicochemical measurements

For each cultivar (Santa Rosa and Sweet Miriam) and developmental stage (S2, S3/S4, S4-I, and S4-II), fruit ethylene (C_2_H_4_ µl kg^−1^ h^−1^), respiration rate (CO_2_ ml kg^−1^ h^−1^), and physicochemical properties were measured on six fruits from each biological replication, as described by Kim *et al.* (2015*a*).

### RNA-Seq analysis

#### RNA-Seq sample preparation

Three biological replicates from Santa Rosa and Sweet Miriam at S2 and S4-II (Table 1) were used to extract high quality RNA using the cetyltrimethylammonium bromide (CTAB)/NaCl method (Chang *et al.*, 1993) with some modifications (Kim *et al.*, 2015*b*). Library analysis, quantification, and paired-end RNA-sequencing (RNA-Seq) were based on Kim *et al.* (2015*b*).

#### Mapping of RNA-Seq reads and gene annotation

Illumina RNA-Seq reads from each replicate were submitted to adaptor trimming and removal of contaminant sequences using fastqc (www.bioinformatics.babraham.ac.uk/projects/fastqc/) and fastx-toolkits (hannonlab.cshl.edu/fastx_toolkit/). The closest well-annotated peach genome (*Prunus persica* version 1.0) provided on Phytozome11 (http://www.phytozome.net/peach.php) was used as primary reference, and the transcriptome fasta was obtained from the genome and annotation (gff3 file) by means of the utility gffread from Cufflinks version 2.1.1 (Trapnell *et al.*, 2010). Transcriptome mapping was achieved by using bowtie2 version 2.1.0 (http://bowtie-bio.sourceforge.net/bowtie2/index.shtml) followed by TopHat2 version 2.0.9 (Trapnell *et al.*, 2009) (https://ccb.jhu.edu/software/tophat/index.shtml) transcript assembling. Gene expression profiles were calculated by FPKM (fragments per kilobase per million reads) methodology (Trapnell *et al.*, 2010). Transcripts with FPKM values above a threshold of 1 FPKM were considered expressed, and thus were kept for further statistical analysis.

Gene annotation was performed using the peach genome (*P. persica* version 1.0) as a reference; therefore, peach gene identifiers as well as their corresponding counterparts from *Arabidopsis thaliana* were obtained using the file PhytozomeV9: Ppersica_139_annotation_info.txt.gz. provided by Phytozome11.

#### Identification of differentially expressed genes (DEGs) from RNA-Seq data

Differential gene expression profiling between Santa Rosa and Sweet Miriam throughout S2 and S4-II was accomplished by using the Cuffdiff version 2.1.1 software package (Cole-Trapnell-lab.github.io/cufflinks/cuffdiff/) with default parameters and cut-offs. DEGs were identified using a 2-fold cut-off in at least one pairwise comparison of the four cultivar–stage combinations (SRS2, SRS4-II, SMS2, and SMS4-II). Statistical significance of the tests was controlled using a *P*-value <0.05. *P*-values were adjusted for multiple testing using the false discovery rate (FDR) method of Benjamini and Hochberg (1995). A DEG was declared as such if the associated *P*-value was <0.05 and *P*_FDR_<0.05 was observed.

The RNA-Seq data used in this study are available at the Sequence Read Archive (SRA, http://www.ncbi.nlm.nih.gov/sra) in the National Center for Biotechnology Information (NCBI) with accession number SRP106354.

#### General characterization of the RNA-Seq data set

**Fig. 1.**
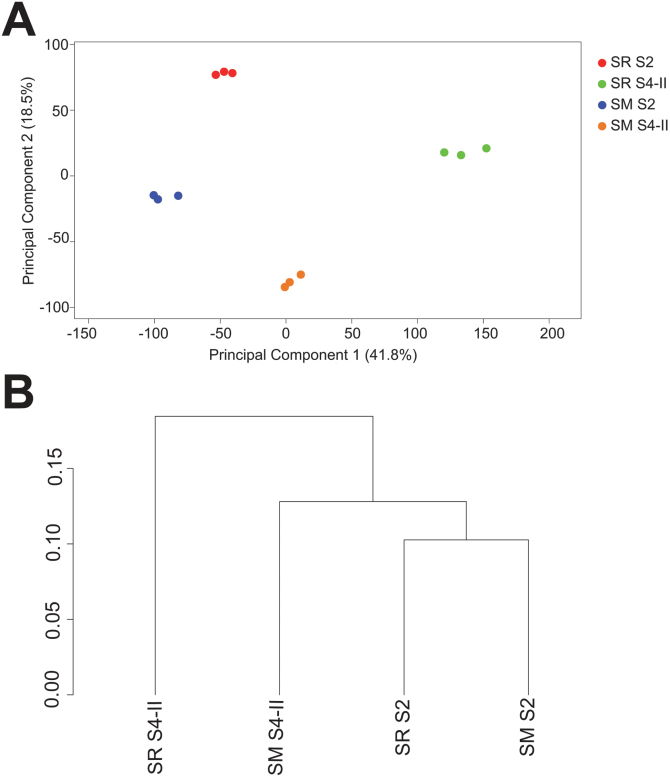
Multivariate analyses of RNA-Seq data obtained from Santa Rosa (SR) and Sweet Miriam (SM) cultivars at two developmental stages (S2 and S4-II). (A) Principal component analysis (PCA) resulting from the expression values of 17 721 genes in each one of the three biological replicates for SR and SM cultivars at S2 and S4-II stages in the RNA-Seq data set. Scores of principal component 1 (*x*-axis) and 2 (*y*-axis) explained 41.8% and 18.5% of total variance, respectively. (B) Cluster dendogram obtained from the mean gene expression level of three biological replicates in each cultivar–stage combination (SRS2, SRS4-II, SMS2, and SMS4-II), from RNA-Seq data.

Principal component analysis (PCA) (Fig. 1A) was performed to obtain a spatial visualization of the whole RNA-Seq data sets to evaluate the expression patterns of each of the four cultivar–stage combinations analyzed (SRS2, SMS2, SRS4-II, and SMS4-II) and to confirm the reliability of our biological replicates. This analysis was achieved by using R 3.3.1 software and the FactoMineR 1.25 package (Husson *et al.*, 2014). Additionally, a cluster dendrogram (Fig. 1B) considering the mean expression values of the three biological replicates for each cultivar–stage combination was created using the CummeRbund’s package (https://www.rdocumentation.org/packages/cummeRbund/versions/2.14.0) .

The Venn diagrams were developed using http://bioinformatics.psb.ugent.be/webtools/Venn/. The fold change in expression levels when comparing among pairs of the cultivar–stage combinations (SMS2/SRS2, SMS4-II/SRS4-II, SRS4-II/SRS2, and SMS4-II/SMS2) of the 5727 DEGs were log2 transformed and used to generate a heat map using the default settings with ‘heatmap.2’ in the ‘gplots’ package of R version 3.3.1 (https://cran.r-project.org/web/packages/gplots/gplots.pdf).

### Pearson correlation coefficient analysis

Pearson correlation coefficients (PCCs) were calculated between Suc, Glu, Fru, G6P, and Sor contents obtained in our previous publication (Kim *et al.*, 2015*a*) and expression levels of the 5727 DEGs obtained from RNA-Seq analysis in this study, in Santa Rosa and Sweet Miriam throughout S2 and S4-II, in pairwise comparisons. The correlation was considered significant if, in at least one pairwise comparison, the PCC was >0.80 (positive correlation) or < –0.80 (negative correlation), at a *P*-value <0.05. All analyses were carried out using function corAndPvalu from the ‘WGCNA’ package (https://labs.genetics.ucla.edu/horvath/CoexpressionNetwork/Rpackages/WGCNA/) using R version 3.3.1 software. Gene Ontology (GO) annotation and literature data were used to obtain sugar metabolism-associated significantly correlated genes.

### Gene co-expression network, module identification, and gene edge number

The construction of the gene co-expression network, followed by module detection of highly correlated genes, was inferred from the 5727 DEGs using weighted gene co-expression network analysis (WGCNA), an R software package (Langfelder and Horvath, 2008). WGCNA network construction and module detection were conducted using default settings. A dynamic cut-tree algorithm was used for automatically and precisely identifying modules in a hierarchical clustering dendogram (Li and Horvath, 2007). GO annotation and literature data were used to obtain all sugar metabolism-related genes within each module. The total number of edges for each sugar metabolism-related gene within each module was estimated using as a cut-off a WGCNA edge weight ≥0.5. Networks were visualized using Cytoscape 3.4.0 (Saito *et al.*, 2012) and Network Analyzer, a Cytoscape plugin.

### Quantification of sugar concentration

Six biological replicates of Santa Rosa and Sweet Miriam fruits and leaves throughout the four developmental stages (S2, S3/S4, S4-I, and S4-II; Table 1) were used to quantify the contents of Suc, Glu, Fru, Sor, G6P, Gal, Raf, Ino, Tre, and the cofactor NAD^+^ through NMR analyses, while Gol contents were quantified through ultra high-performance liquid chromatography-quadrupole time of flight-tandem mass spectrometry (UHPLC-QTOF-MS/MS). Details for these analyses are described in Supplementary data at *JXB* online.

### Real-time quantitative reverse transcription–PCR (qRT–PCR) analysis

RNA was isolated from fruits and leaves of Santa Rosa and Sweet Miriam in S2, S3/S4, S4-I, and S4-II as described above. The sets of primers used for the amplification of the target genes are listed in Supplementary Table S1. Analysis of relative gene expression was performed according to the comparative cycle threshold method as described by Livak and Schmittgen (2001). The expression of the SAND protein-related trafficking protein (MON) was used as a reference as it was validated by our group as one of the best reference genes for precise transcript normalization across different Japanese plum tissue samples and developmental stages (Kim *et al.*, 2015*b*).

### Enzymatic assays

Enzymatic activities of sucrose phosphate synthase (SPS), sucrose synthase (SuSy), cell wall invertases (CWINVs), cytosolic invertase (CytINV), vacuolar invertase (VINV), S6PDH, SOX, and NAD^+^-SDH were determined for fruits and leaves of both cultivars at all stages (Table 1) based on Kim *et al.* (2015*a*). Hexokinase (HK) activity was assayed according to Wiese *et al.* (1999). The Bradford assay (Bradford, 1976) was used for protein quantification using BSA as standard.

### Statistical analysis for fruit ripening patterns, physicochemical measurements, sugar concentration quantification, qRT–PCR, and enzymatic assays

The software package JMP^®^ (ver.10.0, SAS Institute) was used for statistical analyses of the above-mentioned parameters. Two-way ANOVA using Tukey’s test was used to compare between cultivars (Santa Rosa and Sweet Miriam) and developmental stages (S2, S3/S4, S4-I, and S4-II) for significant differences at *P*<0.05 in all cases.

## Results

### Fruit ripening patterns and physicochemical properties of climacteric Santa Rosa and non-climacteric Sweet Miriam bud mutants

Fruits and leaves from two commercial Japanese plum cultivars, a climacteric, early maturing Santa Rosa and its non-climacteric, late maturing bud mutant Sweet Miriam, were collected at four developmental stages (Table 1). The pit hardening stage (S2), occurring after natural fruit drop, was defined by a firmness of ~70 N reached at ~95 and ~115 DAFB in Santa Rosa and Sweet Miriam, respectively (Table 1), as well as by fruit size/weight and skin color values based on previous years’ data (not shown) and our previous study (Kim *et al.*, 2015*a*). During the ripening-related stages, fruit firmness declined from ~40 N to ~20 N, with the Sweet Miriam fruits lagging behind the Santa Rosa fruits by ~42, ~83, and ~100 d, for S3/S4, mature; S4-I, commercial harvest; and S4-II, fully ripe stages, respectively (Table 1). Santa Rosa fruits displayed an increase in respiration rates throughout development, and significantly higher levels of respiration than Sweet Miriam fruits at all four developmental stages (Supplementary Fig. S1A). Respiration burst, typical of its climacteric behavior, was detected between 116 DAFB (S4-I) and 125 DAFB (S4-III) (not shown), similar to what was reported previously (Kim *et al*, 2015*a*). Sweet Miriam displayed a slight increase in respiration rate from S2 (non-ripening stage) to S4-II, with no evident respiration burst (Supplementary Fig. S1A). A significant increase in ethylene production from the mature stage (S3/S4) onwards was detected in Santa Rosa fruits (Supplementary Fig. S1B), while Sweet Miriam maintained significantly lower and constant ethylene production rates throughout all ripening-related stages. Skin and flesh fruit color values, expressed as hue (Hº) angles, changed from green at the early S2 stage to red skin and yellow flesh in both cultivars (Supplementary Fig. S1C, D). Sweet Miriam presented significantly higher soluble solids contents (SSCs) throughout all developmental stages (Supplementary Fig. S1E) and significantly lower titratable acidity (TA) (Supplementary Fig. S1F, G) with respect to Santa Rosa.

### Transcriptome characterization of climacteric Santa Rosa and non-climacteric Sweet Miriam bud mutants

RNA-Seq analysis enabled the identification of 29 096 unique genes (Kim *et al.*, 2015*b*). Subsequent calculation of normalized read counts (FPKM) for each gene resulted in a total of 17 721 genes used for statistical analysis. PCA of the expression values of the 17 721 genes showed that the first two principal components (PC1 and PC2) could explain 60.3% of the total transcript expression level variance in the score plot (Fig. 1A). Each assessed cultivar–stage combination (SRS2, SRS4-II, SMS2, and SMS4-II) was separated in an independent cluster, indicating that there were differences in their expression patterns. Nonetheless, within each cultivar–stage combination cluster, the three biological replicates displayed relatively low variation (Fig. 1A), demonstrating the reproducibility and reliability of the gene expression data sets. A cluster dendogram, built using the mean gene expression level of the three biological replicates in each cultivar–stage combination (SRS2, SRS4-II, SMS2, and SMS4-II) for the 17 721 genes, showed that SRS4-II was relatively distinct from the other cultivar–stage combinations (Fig. 1B). These results suggested that the main differences in gene expression among the cultivars occurred during the ripening phase (S4-II); while, within cultivars, changes in expression throughout development (from S2 to S4-II) showed less variation for Sweet Miriam as compared with Santa Rosa (Fig. 1B).

**Fig. 2.**
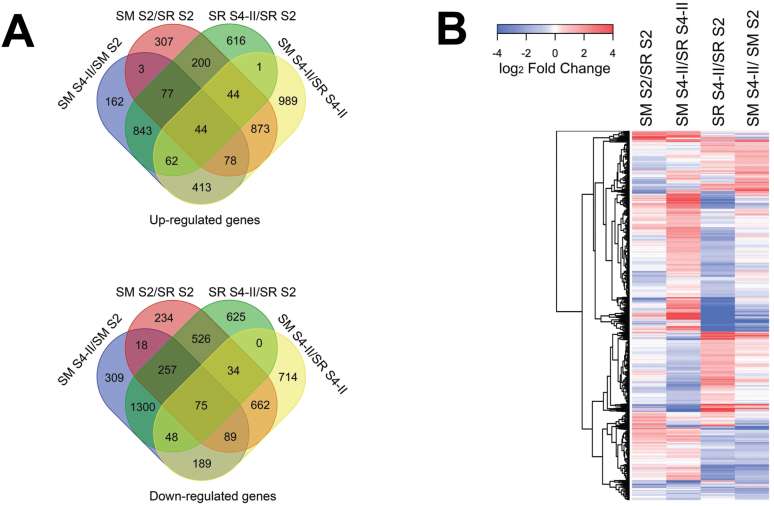
Venn diagram and heatmap analyses of RNASeq data obtained from Santa Rosa (SR) and Sweet Miriam (SM) cultivars at two developmental stages (S2, S4-II). (A) Venn diagram analyses of the 5,727 differentially expressed genes (DEGs), comparing the number of upregulated and downregulated genes within the same stage in different cultivars (SMS2/SRS2 and SMS4-II/SRS4-II) and within the same cultivar in different stages (SRS4-II/SRS2 and SMS4-II/SMS2). (B) Heatmap generated from the 5,727 DEGs from RNA-Seq analysis comparing fold changes in levels of gene expression within the same stage in different cultivars (SMS2/SRS2 and SMS4-II/SRS4-II) and within the same cultivar in different stages (SRS4-II/SRS2 and SMS4-II/SMS2). Heatmap colors represent gene expression fold change levels based on the provided color key scale; red for upregulated genes expression levels, blue for downregulated gene expression levels and white for no changes in gene expression levels.

Differential expression gene profiling of the RNA-Seq data sets, using a 2-fold cut-off value in at least one pairwise comparison of the four cultivar–stage combinations (SRS2, SRS4-II, SMS2, and SMS4-II), identified a total number of 5727 DEGs. When comparing both cultivars at the same developmental stage, the results indicated that during pit hardening (SMS2/SRS2) there were 307 and 234 up-regulated and down-regulated genes, respectively; while at the fully ripe stage (SMS4-II/SRS4-II) there were 989 and 714 up-regulated and down-regulated genes, respectively (Fig. 2A). In addition, when examining the fold change in expression levels, as shown on the heat map (Fig. 2B), less variability was detected in SMS2/SRS2, as compared with SMS4-II/SRS4-II, consistent with the pronounced transcriptomic changes that occurred during ripening of both cultivars (Fig. 2A). On the other hand, when comparing developmental stages within the same cultivar, Santa Rosa (SRS4-II/SRS2) displayed 616 and 625 up-regulated and down-regulated genes, respectively; while Sweet Miriam (SMS4-II/SMS2) exhibited 162 and 309 up-regulated and down-regulated genes, respectively (Fig. 2A). In addition, lower variability within Sweet Miriam fruits was detected during development (SMS4-II/SMS2), as compared with Santa Rosa (SRS4/-II/SRS2) (Fig. 2B). Overall, changes in transcriptomic data, both for the entire gene sets (Fig. 1) and for DEGs (Fig. 2) between the two cultivars, correlated well with the differences in fruit ripening behavior; the relatively similar ethylene-independent S2 stage (pit hardening) (Chalmers and Ende, 1975), followed by ethylene-dependent ripening stages of Santa Rosa fruit, as opposed to ethylene-independent ripening of Sweet Miriam fruits (Kim et al., 2015a).

### Identification of key sugar metabolism-associated genes and their correlation with fruit sugar contents

**Fig. 3.**
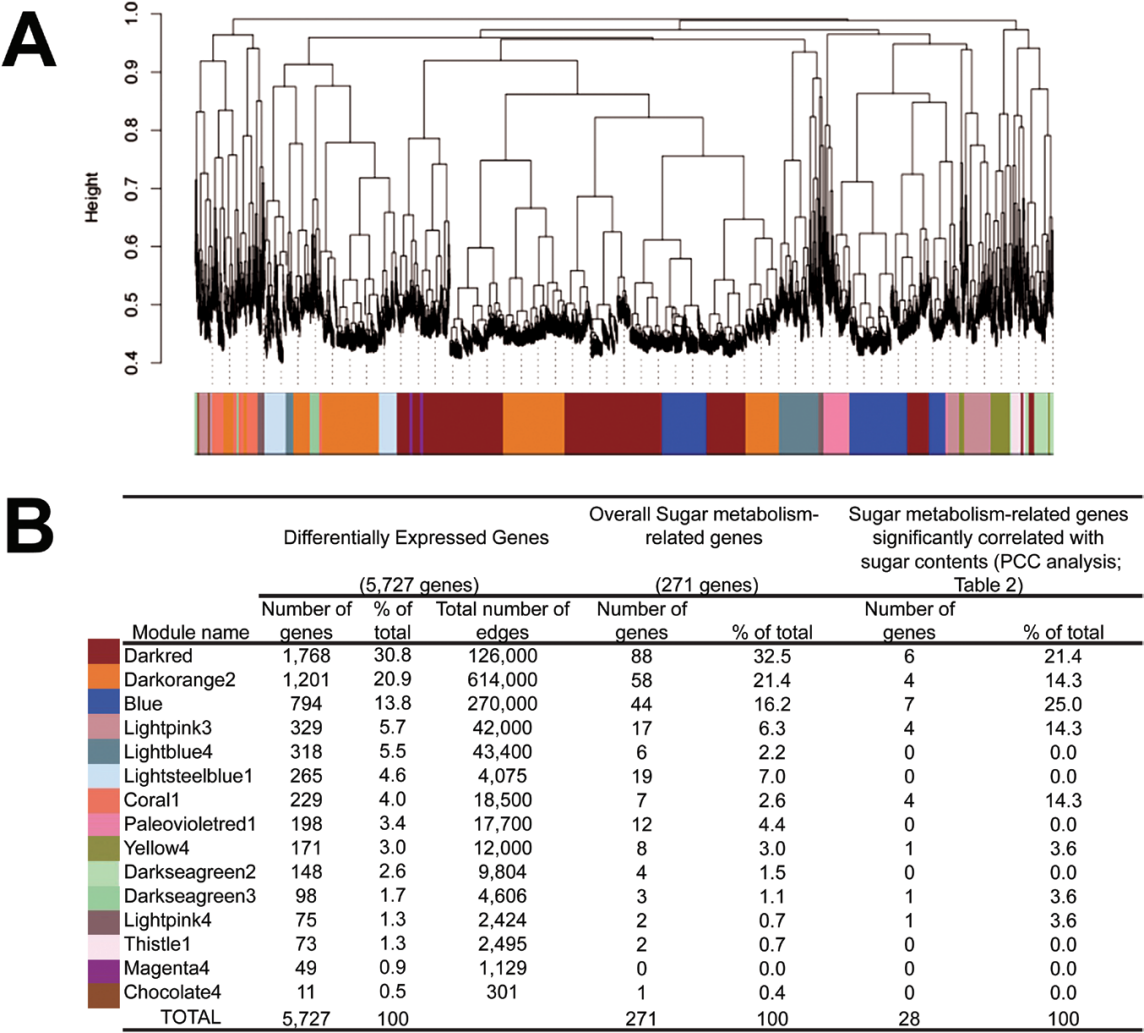
Weighted gene co-expression network analysis (WGCNA) of differentially expressed genes (DEGs) identified from climacteric Santa Rosa (SR) and non-climacteric Sweet Miriam (SM) cultivars throughout two developmental stages. (A) Gene dendogram, obtained by hierarchical clustering, showing 15 identified modules of co-expressed genes among the 5727 DEGs from RNA-Seq data. Each leaf in the tree represents one of the DEGs and each major tree branch represents one of the 15 modules. The lower panel shows the different colors assigned to each one of the modules. (B) Characterization of modules of co-expressed genes. Distribution of all DEGs (including number and percentage of genes as well as number of edges/module) of sugar metabolism genes (including number and percentage of genes/module) and of the 28 sugar metabolism-related genes significantly correlated with sugar contents through Pearson correlation coefficient (PCC) analysis (Table 2) are shown.

A PCC analysis was performed between the contents of the most abundant sugars, Suc, Glu, Fru, Sor, and G6P (Kim *et al.*, 2015*a*), and the expression levels of the 5727 DEGs throughout S2 and S4-II stages, in pairwise PCC comparisons (Supplementary Fig. S2, left panel). The combination of defined GO terms and known sugar metabolism-related genes resulted in 28 sugar metabolism-associated genes that were significantly correlated with sugar contents in at least one pairwise PCC comparison (Table 2). In parallel, WGCNA (Fuller *et al.*, 2007; Langfelder and Horvath, 2008) was performed (Supplementary Fig. S2, right panel). Fifteen WGCNA modules (clusters of highly interconnected genes; Langfelder and Horvath, 2008) labeled by different colors were identified (Fig. 3A). The majority of the 5727 DEGs were grouped into the ‘darkred’ module (30.8%), followed by the ‘darkorange’ and ‘blue’ modules that contained 20.9% and 13.8% of the total DEGs, respectively (Fig. 3B). The other 12 modules comprised 0.5–5.7% of total DEGs (Fig. 3B). The transcriptome analysis identified 271 sugar metabolism-related genes; the majority of these were also grouped into the ‘darkred’ module (32.5%), followed by the ‘darkorange’ and ‘blue’ modules that contained 21.4% and 16.2% of the total sugar metabolism-related genes, respectively (Fig. 3B). The other 12 modules included 0–7% of the total genes (Fig. 3B).

**Table 2.** A list of the sugar metabolism genes significantly correlated with sugar contents A total of 28 genes were identified through Pearson correlation coefficient (PCC) analysis as significantly correlated (in at least one pairwise PCC comparison) with sugar content in fruits of climacteric Santa Rosa and non-climacteric Sweet Miriam cultivars throughout an early (S2) and late (S4-II) stage of development

*Prunus persica* gene IDs	Description of sugar metabolism-related genes	Abbreviation of sugar metabolism-related genes	Sucrose		Glucose		Fructose		Sorbitol		Glucose-6-phosphate	
			PCC	*P*-value	PCC	*P*-value	PCC	*P*-value	PCC	*P*-value	PCC	*P*-value
ppa002625m.g	Cytosolic invertase	CytINV, At-A/N-InvE	0.93	0.0093	0.84	0.0258	0.85	0.0253	–0.59	NS	0.17	NS
ppa004783m.g	*O*-Glycosyl hydrolases family 17 protein	Empty	–0.85	0.0253	–0.90	0.0298	–0.90	0.0290	–0.11	NS	0.48	NS
ppa001535m.g	Sucrose synthase 4	SuSy, AtSuSy4	–0.94	0.0257	0.97	0.0309	0.96	0.0394	–0.26	NS	–0.06	NS
ppa004568m.g	Mevalonate/galactokinase family protein	GALK, GAL1	0.86	0.0245	0.96	0.0291	0.97	0.0293	–0.31	NS	–0.07	NS
ppa002839m.g	β-Galactosidase 16	β-GAL, βGAL16	0.96	0.0093	0.89	0.0107	0.89	0.0117	–0.29	NS	–0.07	NS
ppa005180m.g	UDP-glycosyltransferase superfamily protein	Empty	–0.84	0.0163	–0.92	0.0188	–0.92	0.0188	–0.09	NS	0.46	NS
ppa006549m.g	Glucose-6-phosphate/phosphate translocator-related	APE2,TPT	–0.90	0.0192	0.97	0.0091	0.98	0.0091	–0.30	NS	0.60	NS
ppa001841m.g	Raffinose synthase/seed imbibition 1	RS, AtSIP1,SIP1	0.62	NS	–0.95	0.0151	–0.92	0.0176	0.90	0.0173	–0.92	0.0034
ppa003718m.g	β-Glucosidase 15	β-GLU15	0.65	NS	–0.95	0.0107	–0.94	0.0296	0.97	0.0312	–0.93	0.0273
ppa007869m.g	UDP-glucosyl transferase 71C3	UGT71C3	0.60	NS	–0.97	0.0100	–0.97	0.0333	0.99	0.0081	–0.89	0.0311
ppa005277m.g	UDP-glucosyl transferase 71B6	UGT71B6	0.55	NS	–0.98	0.0132	–0.98	0.0238	0.98	0.0150	–0.88	0.0341
ppa006167m.g	β-Glucosidase 11	β-GLU11	–0.93	0.0347	0.46	NS	0.47	NS	–0.65	NS	0.71	n.s
ppa008264m.g	Galactinol synthase 4	GolS, AtGolS4	–0.85	0.0146	0.26	NS	0.27	NS	–0.47	NS	0.58	NS
ppa007136m.g	α-Galactosidase 2	α-GAL, α-GAL2	0.90	0.0203	0.24	NS	0.25	NS	–0.45	NS	0.62	NS
ppa020483m.g	UDP-glucosyl transferase 76E2	UGT76E2	–0.98	0.0200	0.47	NS	0.47	NS	–0.64	NS	0.77	n.s
ppa005599m.g	Pectin lyase-like superfamily protein	Empty	0.94	0.0194	–0.06	NS	–0.04	NS	0.23	NS	–0.97	0.1331
ppa002559m.g	Glycosyl hydrolase family protein	Empty	0.98	0.0208	–0.44	NS	–0.41	NS	0.56	NS	–0.98	0.0092
ppa000622m.g	Sucrose phosphate synthase	SPS, AtSPS	0.94	0.0320	0.95	0.0280	0.96	0.0241	–0.27	NS	–0.18	NS
ppa003514m.g	Trehalase 1	TRE, AtTRE1	0.97	0.0233	0.93	0.0346	0.94	0.0320	–0.36	NS	–0.09	NS
ppa002511m.g	Cellulose-synthase-like C4	AtCSLC04,ATCSLC4,CSLC04,CSLC4	0.91	0.0277	0.01	NS	0.03	NS	0.15	NS	–0.92	0.0383
ppa008438m.g	UDP-galactose transporter 3	AtUTR3,UTR3	0.86	0.0239	0.06	NS	0.06	NS	0.15	NS	–0.91	0.0149
ppa001730m.g	Raffinose synthase/seed imbibition 2	RS, AtSIP2,SIP2	0.89	0.0208	–0.74	NS	–0.75	NS	0.87	0.0277	–0.85	0.0348
ppa011699m.g	Plant invertase inhibitor superfamily protein	INVINH	0.47	NS	–0.99	0.0057	–0.99	0.0053	0.99	0.0073	–0.81	NS
ppa005294m.g	UDP-glucosyltransferase 74F2	AtSAGT1,GT,SAGT1,SGT1,UGT74F2	–0.06	NS	–0.86	0.0383	–0.88	0.0210	0.90	0.0202	–0.36	NS
ppa004956m.g	UDP-glucosyl transferase 85A2	AtUGT85A2,UGT85A2	0.20	NS	–0.98	0.0021	–0.98	0.0018	0.92	0.0013	–0.63	NS
ppa001889m.g	Cellulose synthase-like B3	ATCSLB03,ATCSLB3,CSLB03	0.38	NS	–0.99	0.0001	–0.99	0.0001	0.97	0.0027	–0.77	NS
ppa002334m.g	Vacuolar invertase	VINV, AtBETAFRUCT4	0.54	NS	0.96	0.0094	0.93	0.0093	0.71	NS	–0.84	0.0157
ppa017606m.g	Sucrose synthase 6	SuSy, AtSuSy6	–0.96	0.0193	0.91	0.0135	0.91	0.0110	–0.31	NS	0.05	NS

NS indicates that the pairwise correlation was not significant.

**Fig. 4.**
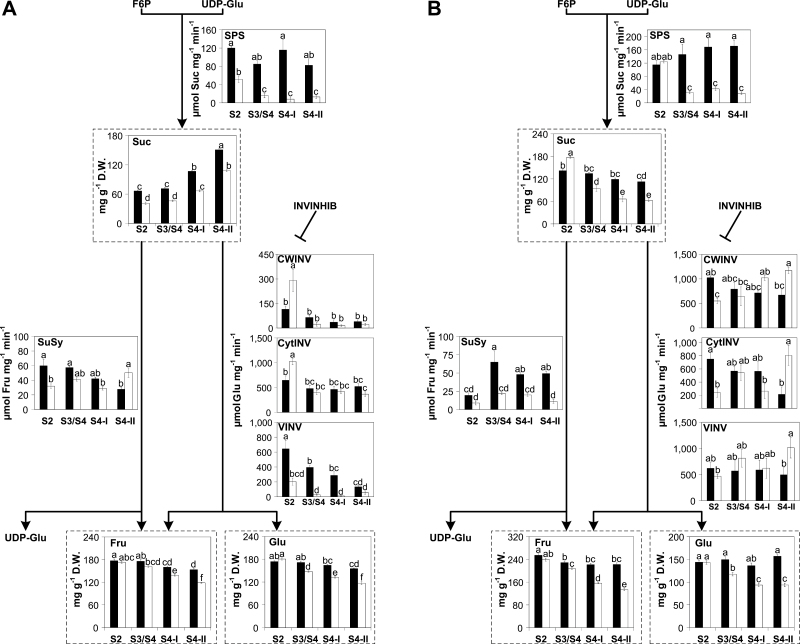
Sucrose metabolism-associated pathways in (A) fruits and (B) leaves of climacteric Santa Rosa (SR; black bars) and non-climacteric Sweet Miriam (SM; white bars) throughout development [S2 (pit hardening)] and ripening [S3/S4 (mature), S4-I (commercial harvest), S4-II (fully ripe)] stages on the tree. Sugar contents are presented in graphs framed by dashed lines and are expressed as milligrams per gram of dry weight (D.W.). Enzyme activities are expressed as moles of metabolite generated/consumed per milligram of protein per unit of time. Values are presented as means ±SE (*n*=6). The data were analyzed using two-way ANOVA followed by Tukey’s test. Different letters indicate significant differences (*P*<0.05). SPS, sucrose phosphate synthase; SuSy, sucrose synthase; CWINV, cell wall invertase; VINV, vacuolar invertase; CytINV, cytosolic invertase; INVINH, invertase inhibitor; Suc, sucrose; Fru, fructose; Glu, glucose; F6P, fructose-6-phosphate; UDP-Glu, UDP-glucose.

Approximately half of the 28 sugar metabolism-related genes identified through PCC analysis (Table 2) were grouped in the ‘blue’ (25%) and ‘darkred’ (21.4%) modules, while the ‘darkorange2’, ‘lightpink3’, and ‘coral1’ each contained 14.3% of the genes (Fig. 4B). Modules ‘lightpink4’, ‘yellow4’, and ‘darkseagreen3’ each included 3.6% of the total highly correlated genes (Fig. 3B). Furthermore, the ‘darkorange2’ module showed the highest number of edges between the genes in the subnetwork (614 000) (Fig. 3B), followed by the ‘blue’ and ‘darkred’ modules, with 270 000 and 126 000 edges, respectively. Since genes showing the highest number of edges were considered as hub genes (Hollender *et al.*, 2014), we further determined the number of edges of each of the 271 sugar metabolism-related genes (identified from the DEGs by GO terms and published data), within each module (Supplementary Fig. S2, right panel). Genes within each module were ranked from largest to smallest based on their number of edges (Supplementary Table S2).

By combining results obtained from PCC analysis and WGCNA (Supplementary Fig. S2), we were able to identify 11 key sugar metabolism-associated genes significantly correlated with sugar contents and showing the highest number of edges within their respective modules (Table 3; Supplementary Table S2). Among these 11 genes, 5 were related to Suc metabolism (sucrose synthase, *SuSy*; sucrose phosphate synthase, *SPS*; cytosolic invertase, *CytINV*; vacuolar invertase, *VINV*; and invertase inhibitor, *INVINH*), while the other 6 genes were related to Gal (α-galactosidase, *α-GAL*; β-galactosidase, *β-GAL*; and galactokinase, *GALK*), Tre (trehalase, *TRE*), Gol (galactinol synthase, *GolS*) and Raf (raffinose synthase, *RS*) metabolism (Table 3).

**Table 3.** A list of the 11 key sugar metabolism genes A total of 11 key genes were identified for further targeted validation analyses due to their significant correlation with sugar contents as identified through Pearson correlation coefficient (PCC) analysis (Table 2) and to their high number of edges within their respective modules, suggesting that they could behave as putative hub genes, as revealed by weighted gene co-expression network analysis (Supplementary Table S2)

*Prunus persica* gene IDs	Description of sugar metabolism-related genes	Abbreviation of sugar metabolism-related genes	Module belongness	No. of connections in network
ppa002625m.g	Cytosolic invertase	*CytINV*	blue	67
ppa002839m.g	β-Galactosidase	*β-GAL*	blue	194
ppa004568m.g	Galactokinase	*GALK*	blue	188
ppa001841m.g	Raffinose synthase	*RS*	coral1	11
ppa007136m.g	α-Galactosidase	*α-GAL*	darkorange2	136
ppa008264m.g	Galactinol synthase	*GolS*	darkorange2	111
ppa000622m.g	Sucrose phosphate synthase	*SPS*	darkred	34
ppa003514m.g	Trehalase	*TRE*	darkred	58
ppa011699m.g	Invertase Inhibitor	*INVINH*	lightpink3	20
ppa002334m.g	Vacuolar Invertase	*VINV*	lightpink4	11
ppa017606m.g	Sucrose synthase	*SuSy*	yellow4	26

### Functional validation of sugar metabolism-associated genes and their related metabolites

A comprehensive validation of the sugar metabolism-associated genes (Table 3) and their related metabolites was carried out using qRT–PCR and enzymatic assays of Santa Rosa and Sweet Miriam fruits and leaves during pit hardening (S2) and throughout three ripening stages (S3/S4, S4-I, and S4-II) (Table 1; Supplementary Fig. S2). Given the significant increase in Sor content in Sweet Miriam fruits (see below), genes associated with Sor metabolism were also included in our analysis.

#### Suc, Glu, and Fru metabolism

In Santa Rosa and Sweet Miriam cultivars, Suc contents gradually increased in fruits and decreased in leaves throughout development (Fig. 4). Suc contents in Santa Rosa fruits were higher than those in Sweet Miriam fruits during all analyzed stages (Fig. 4A). In leaves, except at the pit hardening stage, Santa Rosa displayed higher Suc contents than Sweet Miriam (Fig. 4B). SPS (EC 2.4.1.14) activity was higher in Santa Rosa than Sweet Miriam fruits and leaves (except in leaves at S2) and remained constant throughout ripening in Santa Rosa, while SPS activity decreased in Sweet Miriam fruits and leaves (Fig. 4). *SPS* transcript levels in Santa Rosa and Sweet Miriam fruits and leaves displayed similar patterns to the SPS activities (Fig. 4; Supplementary Fig. S3). Santa Rosa fruits and leaves displayed higher SPS activities and *SPS* expression levels with respect to Sweet Miriam (Fig. 4; Supplementary Fig. S3).

In general, Glu and Fru contents of fruits and leaves from both cultivars decreased towards the fully ripe stage, with Sweet Miriam showing a steeper decrease (Fig. 4). Glu and Fru contents of fruits and leaves were higher in Santa Rosa throughout the ripening stages (Fig. 4).

Suc catabolism can be mediated by the action of SuSy (EC 2.4.1.13) and invertases (EC 3.2.1.26). Although SuSy could also facilitate the reverse reaction, in fruits and leaves SuSy mediates the breakdown of Suc into UDP-glucose and Fru (Miron and Schaffer, 1991; Klann *et al.*, 1993; Yamaki, 1994; Kleczkowski *et al.*, 2010). Both *SuSy* expression and activity decreased in Santa Rosa fruits towards the fully ripe stage, while in Sweet Miriam fruits its activity increased from S2 to S4-II stages (Fig. 4A; Supplementary Fig. S3A). In Santa Rosa leaves, SuSy activity increased towards S4-II and remained constant in Sweet Miriam (Fig. 4B). Three types of invertase activities (CytINV, VINV, and CWINV), cleaving Suc to Glu and Fru (Li *et al.*, 2012; Sturm, 1999), were detected in fruits and leaves from both cultivars. In fruits, CytINV and VINV activities were higher than that of CWINV (Fig. 4A). In general, all fruit invertase activities were high at the S2 stage and decreased towards the fully ripe stage (Fig. 4A). In leaves, similar levels of CWINV, CytINV, and VINV activities were detected (Fig. 4B). In Santa Rosa leaves, CWINV and VINV remained unchanged during leaf development while CytINV decreased (Fig. 4B). In Sweet Miriam leaves, CWINV, CytINV, and VINV activities increased during ripening. Since invertase activities are regulated through post-translational suppression via the action of invertase inhibitors (INVINHs) (Jin *et al.*, 2009), we assessed *INVINH* transcript levels in both fruits and leaves (Supplementary Fig. S3). In fruits, *INVINH* transcript levels were similar in Santa Rosa and Sweet Miriam and increased towards the fully ripe stage (Supplementary Fig. S3A). In Santa Rosa leaves, *INVINH* transcript levels were very low as compared with Sweet Miriam (Supplementary Fig. S3B). Sweet Miriam leaves displayed 10-fold higher *INVINH* transcripts during early and mature developmental stages, but decreased to levels similar to Santa Rosa leaves towards the fully ripe stage (Supplementary Fig. S3B).

#### Sor metabolism

**Fig. 5.**
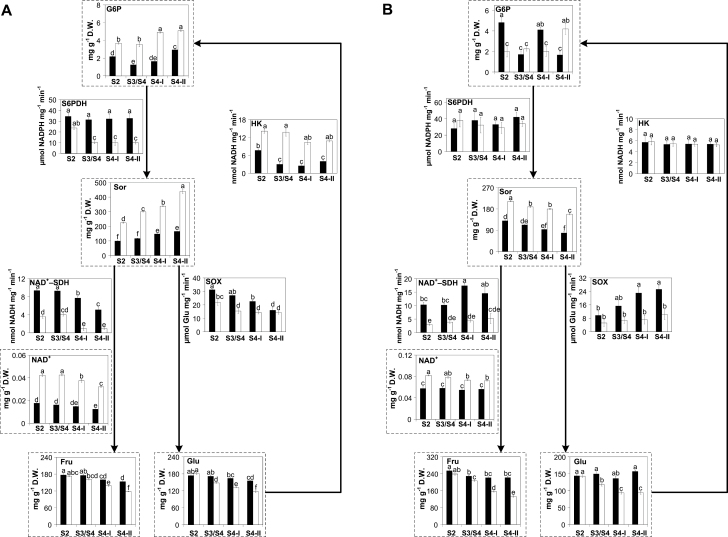
Sorbitol metabolism-associated pathways in (A) fruits and (B) leaves of climacteric Santa Rosa (SR; black bars) and non-climacteric Sweet Miriam (SM; white bars) throughout development [S2 (pit hardening)] and ripening [S3/S4 (mature), S4-I (commercial harvest), S4-II (fully ripe)] stages on the tree. Sugar contents are presented in graphs framed by dashed lines and are expressed as milligrams per gram of dry weight (D.W.). Enzyme activities are expressed as moles of metabolite generated/consumed per milligram of protein per unit of time. Values are presented as means ±SE (*n*=6). The data were analyzed using two-way ANOVA followed by Tukey’s test. Different letters indicate significant differences (*P*<0.05). S6PDH, sorbitol-6-phosphate dehydrogenase; NAD^+^-SDH, NAD^+^-dependent sorbitol dehydrogenase; SOX, sorbitol oxidase; HK, hexokinase; G6P, glucose-6-phosphate; Sor, sorbitol; Fru, fructose; Glu, glucose.

Similar to Suc (Fig. 4), Sor contents increased in fruits and decreased in leaves of both cultivars during development (Fig. 5). Notably, Sor contents of Sweet Miriam fruits and leaves were much higher than those in Santa Rosa at all stages (Fig. 5). Sor synthesis is mediated by the activity of S6PDH (EC 1.1.1.200), which reduces G6P to sorbitol-6-phosphate (Teo *et al.*, 2006; Suzuki and Dandekar, 2014; Suzuki, 2015). S6PDH activity (measured as µmol of NADPH mg^−1^ min^−1^ produced; thus the higher the amounts of NADPH produced the lower the S6PDH activity) increased towards the fully ripe stage in Sweet Miriam, while it remained unchanged in Santa Rosa fruits (Fig. 5A). The high Sor contents and increased S6PDH activity were well correlated with the elevated *S6PDH* transcripts in Sweet Miriam fruits (Supplementary Fig. S4A). In fruits from both cultivars, G6P contents increased throughout the ripening-related stages (Fig. 5A), while Santa Rosa leaves displayed a reduction towards the S4-II stage and Sweet Miriam showed increased G6P contents (Fig. 5B). Hexokinase (HK; EC 2.7.1.1) activity, that phosphorylates Glu to G6P (Li *et al.*, 2012), decreased in Santa Rosa fruits throughout the four assayed stages, while it remained unchanged in Sweet Miriam fruits (Fig. 5A). HK activity was 2- to 4-fold higher in Sweet Miriam than in Santa Rosa fruits (Fig. 5A). In leaves, enzymatic activity and mRNA levels were constant throughout the four assayed stages in both cultivars (Fig. 5B; Supplementary Fig. S4B).

Degradation of Sor is mediated by the action of NAD^+^-SDH (EC 1.1.1.14) into Fru using NAD^+^ as a cofactor, and through SOX (EC1.1.3.x) into Glu (Bianco and Rieger, 2002; Teo *et al.*, 2006). In fruits from both cultivars, NAD^+^-SDH and SOX activities decreased towards the fully ripe stage (Fig. 5A) and increased only in Santa Rosa leaves (Fig. 5B). Nevertheless, NAD^+^-SDH and SOX activities were 2- to 4-fold higher in Santa Rosa fruits and leaves as compared with Sweet Miriam, and this was paralleled by lower NAD^+^ levels in fruits and leaves of Santa Rosa (Fig. 5).

#### Metabolism of minor sugars

**Fig. 6.**
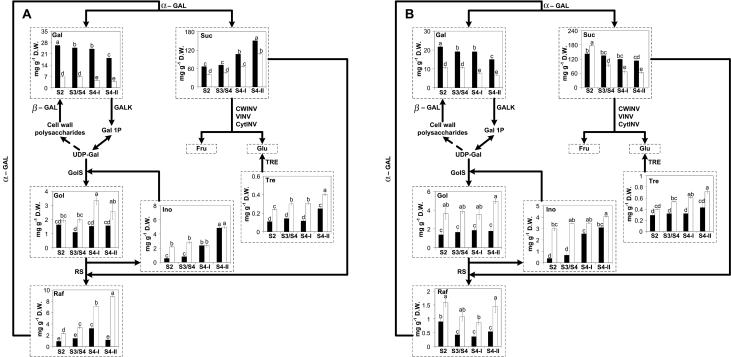
Minor sugar metabolism-associated pathways. Galactose, galactinol, raffinose, myo-inositol, and trehalose metabolism-associated pathways in (A) fruits and (B) leaves of climacteric Santa Rosa (SR; black bars) and non-climacteric Sweet Miriam (SM; white bars) throughout development [S2 (pit hardening)] and ripening [S3/S4 (mature), S4-I (commercial harvest), S4-II (fully ripe)] stages on the tree. Sugar contents are presented in graphs framed by dashed lines and are expressed as milligrams per gram of dry weight (D.W.). Values are presented as means ±SE (*n*=6). The data were analyzed using two-way ANOVA followed by Tukey’s test. Different letters indicate significant differences (*P*<0.05). GALK, galatokinase; α-GAL, α-galactosidase); β-GAL, β-galactosidase; GolS, galactinol synthase; RS, raffinose synthase; TRE, trehalase; CWINV, cell wall invertase; VINV, vacuolar invertase; CytINV, cytosolic invertase; Gal, galactose; Gol, galactinol; Raf, raffinose; Ino, myo-inositol; Tre, trehalose; Fru, fructose; Glu, glucose; Suc, sucrose; Gal 1P, galactose-1-phosphate; UDP-Gal, UDP-galactose.

Gal contents in fruits and leaves decreased towards the fully ripe stage in both cultivars yet Gal contents in Santa Rosa were higher than those of Sweet Miriam (Fig. 6). Gal synthesis via α-GAL (EC 3.2.1.22) results from the hydrolysis of Raf to yield free Gal and Suc (Hubbard *et al.*, 1989; Dai *et al.*, 2006). In fruits and leaves from both cultivars, *α-GAL* mRNA levels decreased towards the fully ripe stage, except for Sweet Miriam fruits where these remained unchanged (Supplementary Fig. S5). Additionally, the cleavage of galactosyl residues from cell wall polysaccharides via β-GAL (EC 3.2.1.23) could also contribute to the increase in the free Gal pool (Sozzi *et al.*, 1998). *α-GAL* and *β-GAL* transcripts were higher in Santa Rosa fruits and leaves than those in Sweet Miriam (Supplementary Fig. S5) and correlated well with the higher Santa Rosa Gal contents (Fig. 6). Gal can also be phosphorylated by GalK (EC 2.7.1.6) into Gal 1-P which is converted to UDP-galactose (UDP-Gal) possibly by a pyrophosphorylase (Dai *et al.*, 2006). *GalK* mRNA levels increased in Santa Rosa fruits and decreased in Sweet Miriam fruits throughout development, and were higher in Santa Rosa fruits (Supplementary Fig. S5A). In leaves, *GalK* transcripts remained unchanged in both cultivars, with Sweet Miriam displaying higher transcripts levels (Supplementary Fig. S5B). On the other hand, UDP-Gal together with Ino are used as substrates by GolS (EC 2.4.1.123) for the synthesis of Gol (Nishizawa *et al.*, 2008). During development and ripening, Gol contents remained constant in Santa Rosa fruits and leaves while Gol increased in Sweet Miriam fruits, but not in leaves (Fig. 6). *GolS* transcripts in Sweet Miriam fruits and leaves, paralleled the increased Gol contents observed in this cultivar (Supplementary Fig. S5). Gol, together with Suc, form Raf through the action of RS (EC 2.4.1.82), releasing Ino (Pillet *et al.*, 2012). *RS* displayed increasing mRNA levels towards the fully ripe stage in fruits from Santa Rosa and Sweet Miriam and only in leaves from Sweet Miriam, while Santa Rosa remained unchanged (Supplementary Fig. S5). Accordingly, Raf contents increased in Sweet Miriam but not in Santa Rosa fruits (Fig. 6A) and Raf contents in Sweet Miriam leaves were higher than those in Santa Rosa (Fig. 6B). Tre contents increased in fruits from both cultivars towards the fully ripe stage and in Sweet Miriam leaves (Fig. 6). Catabolism of Tre is mediated by the action of TRE (3.2.1.28) (Ponnu *et al.*, 2011). *TRE* mRNA levels remained unchanged in fruits from both cultivars during fruit development and ripening, while it decreased in leaves (Supplementary Fig. S5).

## Discussion

**Fig. 7.**
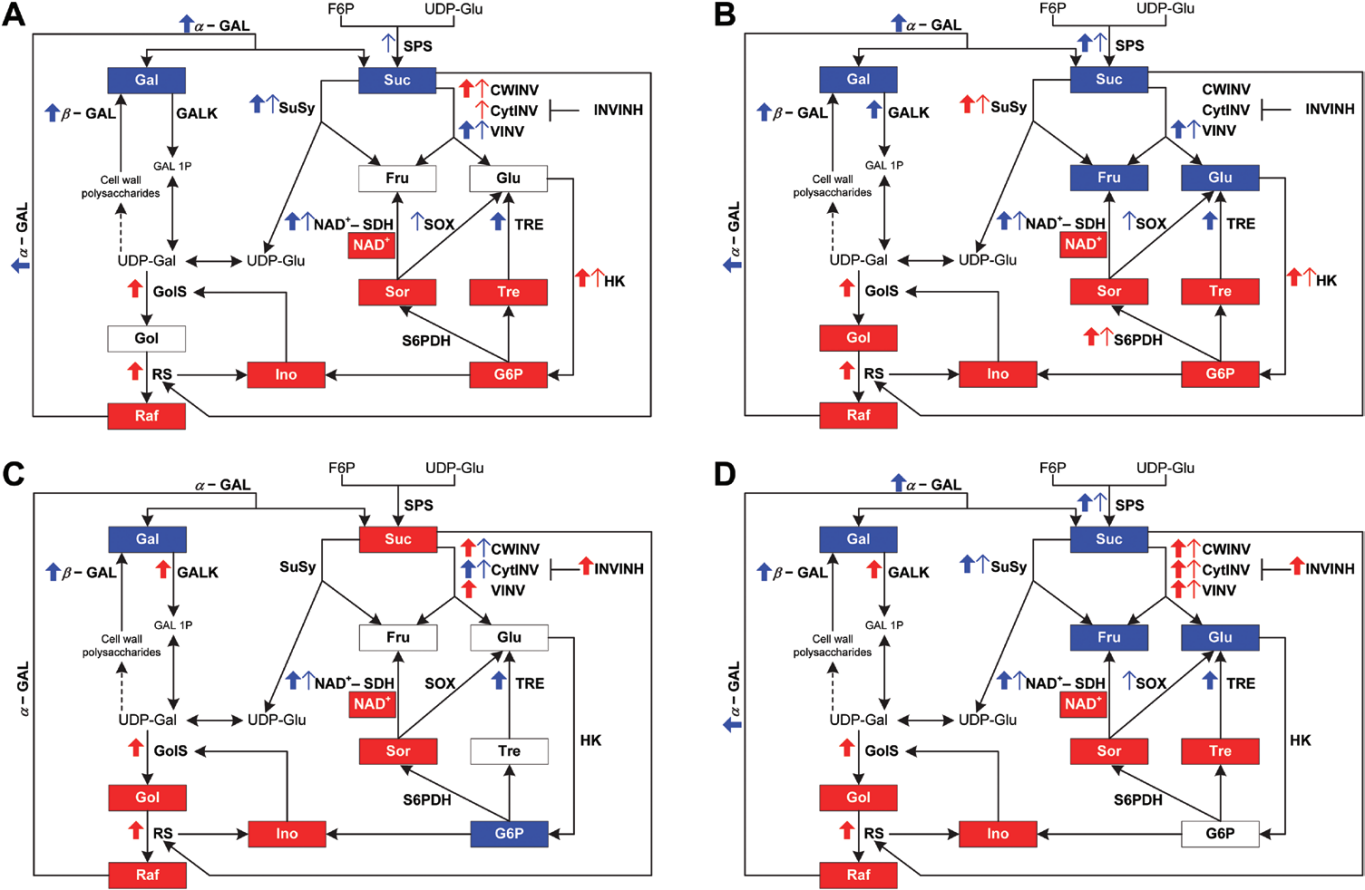
Schematic summary of the overall comparison of sugar metabolism-related pathways in leaves and fruits of climacteric Santa Rosa (SR) and non-climacteric Sweet Miriam (SM) plum cultivars. Sugar metabolism-related pathways are presented for leaves and fruits of SR and SM cultivars during an early stage of development (S2: pit hardening: A, fruits and C, leaves) and throughout ripening [(S3/S4 (mature), S4-I (commercial harvest) and S4-II (fully ripe): B, fruits and D, leaves)]. Sugars are presented in boxes that when colored blue or red indicate that a specific sugar presented a significantly higher content in SR and SM, respectively. Thick upward blue and red arrows indicate mRNA levels that were significantly higher in SR and SM, respectively. Thin upward blue and red arrows represent a significantly higher activity level in SR and SM, respectively. SPS, sucrose phosphate synthase; SuSy, sucrose synthase; CWINV, cell wall invertase; VINV, vacuolar invertase; CytINV, cytosolic invertase; INVINH, invertase inhibitor; S6PDH, sorbitol-6-phosphate dehydrogenase; NAD^+^-SDH, NAD^+^-dependent sorbitol dehydrogenase; SOX, sorbitol oxidase; HK, hexokinase; GALK, galactokinase; α-GAL, α-galactosidase; β-GAL, β-galactosidase; GolS, galactinol synthase; RS, raffinose synthase; TRE, trehalase; Suc, sucrose; Fru, fructose; Glu, glucose; F6P, fructose-6-phosphate; UDP-Glu, UDP-glucose; G6P, glucose-6-phosphate; Sor sorbitol; Gal, galactose; Gol, galactinol; Raf, raffinose; Ino, myo-inositol; Tre, trehalose; Gal 1P, galactose-1-phosphate; UDP-Gal, UDP-galactose.

In this study, we compared biochemical and molecular characteristics of two genetically related Japanese plums (*Prunus salicina* Lindl.). While plum fruits, in general, and the cultivar Santa Rosa, in particular, have been historically classified as climacteric, its bud sport mutant Sweet Miriam (Minas *et al.*, 2015) is a non-climacteric fruit (Kim *et al.*, 2015a). The existence of two contrasting ripening behaviors in fruits of the same genetic background offers a unique experimental system to investigate the influence of ethylene on sugar metabolism and fruit sugar composition. Although the expression of genes associated with sugar metabolism in fruits has been extensively studied in a number of climacteric and non- climacteric species (Borsani *et al.*, 2009; Dai *et al.*, 2011; Li *et al.*, 2012; Beauvoit *et al.*, 2014; Desnoues *et al.*, 2016; D.-G. Hu *et al.*, 2016), studies comparing global gene expression, enzymatic activities, and sugar contents in climacteric versus non-climacteric species/cultivars, and additionally including leaves, are scanty (Osorio *et al.*, 2012). Our initial study revealed altered sugar metabolism and differences in sugar contents between the fruits of Santa Rosa and Sweet Miriam cultivars (Kim *et al.*, 2015*a*). Non-climacteric fruits displayed enhanced sorbitol contents, and a link between ethylene and fruit sugar homeostasis was suggested (Kim *et al.*, 2015*a*). Here, we used transcriptomics and metabolomics to identify genes associated with the notable differences in sugar composition between the climacteric and non-climacteric fruits. Additionally, WGCNA allowed the identification of a number of sugar metabolism-associated genes that could act as putative hubs (Langfelder and Horvath, 2008). We functionally assessed the role of these genes and demonstrated their involvement in the differences in sugar metabolism between fruits with contrasting ripening behaviors. A schematic display illustrating the overall summary of the results obtained in this work is presented in Figure 7.

### Non-climacteric fruit ripening and changes in fruit sugar homeostasis

A number of studies suggested a possible relationship between sugar and ethylene metabolism. Suc stimulated ethylene production in tomato through increased expression of genes associated with ethylene biosynthesis and signaling (Li *et al.*, 2016), and, in strawberry, exogenous Suc accelerated fruit ripening (Jia *et al.*, 2013). Our previous results (Kim *et al.*, 2015*a*) showed increased Sor contents of non- climacteric Sweet Miriam fruits, suggesting a reciprocal correlation between ethylene and Sor. This notion appeared to be supported by the high Sor contents of climacteric apple fruits treated with the inhibitor of ethylene perception, 1-methylcyclopropene (1-MCP) (Lee *et al.*, 2012) and by the decrease in the protein levels of S6PDH, catalyzing Sor synthesis, in ethylene-treated apple fruits (Zheng *et al.*, 2013).

The gradual decrease in Sor and Suc leaf contents during development and the parallel increase in the fruits of both Santa Rosa and Sweet Miriam cultivars (Figs 4, 5) were in good agreement with the role of Sor and Suc as the major photoassimilates translocated from leaves to fruits in members of the *Rosaceae* family (Bieleski, 1982; Li *et al.*, 2012). In general, changes in Sor contents during fruit development and the Sor increase seen in Sweet Miriam fruits were well correlated with the high levels of G6P and higher S6PDH activity and *S6PDH* transcripts (Figs 5A, 7A, B; Supplementary Fig. S4A). Free Suc contents are determined by the balance between Suc synthesis (via SPS) and Suc degradation (via the action of SuSy and invertases). The relatively high *SPS* expression levels and SPS activity in Santa Rosa leaves and fruits correlated well with the higher Suc contents (Figs 4, 7; Supplementary Fig. S3). This is consistent with Hubbard *et al.* (1991) who reported that SPS activity within the fruit itself is an important contributor to fruit Suc contents. Supporting this notion, reports in other climacteric fruits, such as tomato (Carrari *et al.*, 2006; Steinhauser *et al.*, 2010), melons (Dai *et al.*, 2011), jackfruit (L. Hu *et al.*, 2016), and apples (Li *et al.*, 2012), demonstrated the importance of SPS in sucrose accumulation. With regards to Suc degradation, at least during the S2 stage, CWINV and CytINV activities were lower in Santa Rosa than in Sweet Miriam fruits, while VINV and SuSy were higher in Santa Rosa fruits (Figs 4A, 7A). As overall invertases activities were considerably higher than SuSy activities, and assuming that the extractable activities of these enzymes in the total protein extracts paralleled their *in vivo* activities, our results would suggest that invertases provided the major route for Suc breakdown, as suggested in peach and tomato (Klann *et al.*, 1993; Bianco *et al.*, 1999; Nonis *et al.*, 2007). Moreover, the reduction in invertase activities in both cultivars throughout the ripening-related stages, in agreement with the increase in transcript levels of *INVINH* (Figs. 4, 7; Supplementary Fig. S3) was also observed in peach (Vizzotto *et al.*, 1996; Bianco *et al.*, 1999) underlining the key role invertases play in determining overall fruit sugar composition (Bianco *et al.*, 1999).

During ripening, Glu and Fru contents were higher in Santa Rosa fruits (Figs 4A, 7B). Although VINV activity could be contributing to the higher hexose amounts in Santa Rosa fruits, the enhanced Sor breakdown in Santa Rosa fruits was a significant source of Glu and Fru in Santa Rosa. This conclusion is supported by the higher NAD^+^-SDH and SOX enzymatic activities and *NAD*^*+*^*-SDH* transcripts, and lower NAD^+^ levels in Santa Rosa fruits (Figs 5A, 7B; Supplementary Fig. S4A) and by the report showing that NAD^+^-SDH is the key enzyme determining Fru concentrations in peach fruits (Kanayama *et al.*, 2005). An additional observation supporting the above-described scenario comes from both the *HK* transcript levels and HK enzyme activity. HK, which phosphorylates Glu into G6P, the precursor of Sor, displayed both lower activity and low *HK* transcript levels in Santa Rosa than in Sweet Miriam fruits (Figs 5A, 7B; Supplementary Fig. S4A). In apples, a decreased HK activity was observed throughout ripening, suggesting a lower glucose metabolism (Li *et al.*, 2012; Zhao *et al.*, 2016) similar to what was observed in Santa Rosa fruits.

### Sugar metabolism in leaves

In leaves, Santa Rosa’s Suc contents were lower than in Sweet Miriam during the S2 stage (Figs 4B, 7C). These results could be explained by the higher CWINV and CytINV activities, associated with the low *INV-INH* transcript levels in Santa Rosa leaves (Figs 4B, 7C; Supplementary Fig. S3B). The lack of increased Glu and Fru contents (Figs 4B, 7C) can be explained by the translocation of hexoses from leaves into fruits, where they are re-converted to sucrose (Moing *et al.*, 1987). Differences in leaf SPS activity and transcript levels between the two cultivars were in good agreement with the differences in sucrose contents during ripening (Figs 4B, 7D; Supplementary Fig. S3B). Also, the transcripts and activities of CWINV, CytINV, and VINV, at least at the fully mature stage, were higher in Sweet Miriam, in agreement with the low Suc contents in this cultivar (Fig. 4B; Supplementary Fig. S3B). The higher SuSy transcripts and activity in Santa Rosa leaves could be contributing to the high Fru contents (Figs 4B, 7D; Supplementary Fig. S3B) as a result from a shift towards an enhanced Sor cleavage.

### The non-climacteric behavior induces changes in UDP-Gal and trehalose metabolism in the fruits

A noteworthy difference between Santa Rosa and Sweet Miriam fruits was their altered UDP-Gal metabolism. These differences were indicated by the higher levels of the polyols Gol and Ino and the oligosaccharide Raf, and the lower contents of Gal in Sweet Miriam (Figs 6, 7). UDP-Gal can be targeted towards cell wall biosynthesis through galactosyltransferases (Oomen *et al.*, 2004; Seifert, 2004; Barber *et al.*, 2006), can be used as a substrate for Suc synthesis through its interconversion to UDP-Glu (Aizat *et al.*, 2014), or can be used as a substrate for Raf biosynthesis via GolS (Gangl *et al.*, 2015; Handley *et al.*, 1983). Our results suggested that in Santa Rosa, UDP-Gal was targeted towards Gal, while in Sweet Miriam it was targeted towards Raf (Figs 6, 7). In Santa Rosa, these results were probably a consequence of the increased cleavage of galactosyl residues from cell wall polysaccharides through β-GAL, especially during the fruit softening stage (Gross, 1985; Oomen *et al.*, 2004), and increased cleavage of Raf into Gal and Suc through α-GAL (Fig. 7; Supplementary Fig. S5). In Sweet Miriam fruits and leaves, UDP-Gal seemed to be targeted towards Raf biosynthesis via increased *GolS* and *RS* transcript levels, as overall Raf contents were higher in Sweet Miriam (Figs 6, 7; Supplementary Fig. S5). In addition, Ino, a substrate for GolS and released by RS (Pillet *et al.*, 2012), provides a cycle which was overall increased in Sweet Miriam as compared with Santa Rosa (Figs 6, 7).

What might be the roles of the elevated contents of Gal in Santa Rosa and Gol, Raf, and Ino in Sweet Miriam? Free Gal, higher in Santa Rosa fruits, has been shown to increase ethylene production and induce earlier ripening in mature green tomatoes (Gross, 1985). This promotion of ripening due to the Gal-induced increase in ethylene production has been associated with the stimulation of 1-aminocyclopropane-1-carboxylic acid synthase (ACS) activity, the rate-limiting step in ethylene biosynthesis, as well as to a transient increase in 1-aminocyclopropane-1-carboxylic acid (ACC) (Kim *et al.*, 1987). Thus, the higher levels of Gal in Santa Rosa (Figs 6, 7) would suggest a link with the climacteric behavior of this cultivar, that could be further explored. Regarding Gol, Raf, and Ino, several reports have indicated that these metabolites are associated with protection against stresses due to their high antioxidant capacities (Taji *et al.*, 2002; Xue *et al.*, 2007; Nishizawa *et al.*, 2008; Valluru and Van den Ende, 2011 ). The fruit ripening process comprises a series of oxidative activities (Meir *et al.*, 1991; Rogiers *et al.*, 1998; Jimenez *et al.*, 2002), specifically during cell wall breakdown and overall fruit softening (Airianah *et al.*, 2016). Gol and Raf were dramatically elevated in peach fruits exposed to heat and cold stresses during post-harvest storage (Lurie and Crisosto, 2005). Therefore, it is possible that the higher contents of Gol, Ino, and Raf in Sweet Miriam fruits (Figs 6, 7) improve their ability to cope with the oxidative processes occurring during ripening, as suggested by Aizat *et al.* (2014) in non-climacteric *Capsicum*. During ripening in post-harvest storage, these compounds were also higher in Sweet Miriam fruits, supporting their role as oxidative stress protectants (M. Farcuh *et al*., unpublished results). In addition to Gol, Ino, and Raf, Sweet Miriam fruits and leaves also displayed higher Tre contents, that were well correlated with lower TRE transcript levels (Figs 6, 7; Supplementary Fig. S5). Tre has also been reported to play signaling/regulatory roles in plant stress responses (Patrick *et al.*, 2013), suggesting that the increase in Tre contents in Sweet Miriam could also contribute to cope with ripening-associated oxidative stress conditions.

While in our previous publication we concentrated on characterizing sugar contents and some of their corresponding enzymes using two phenological stages and only in fruits, here we used a systems biology approach, combing gene expression, metabolomics, and biochemical analyses to show a reprograming of metabolism of major and minor sugars occurring in fruits and leaves of a non-climacteric bud mutant plum cultivar at four developmental stages. Non-climacteric plums accumulated higher amounts of Sor and lower amounts of Suc, Glu, and Fru than climacteric plums, and the higher amounts of Sor were a consequence of both increased synthesis, mediated by S6PDH, and decreased breakdown, mediated by NAD^+^-SDH and SOX. The non-climacteric behavior was also associated with a shift of UDP-Gal metabolism towards Raf and Gol, as well as the increase in Tre, probably playing a role in improving the overall ability of non-climacteric fruits to cope with oxidative processes associated with fruit ripening. The lower Gal contents in Sweet Miriam could also play a role in its non-climacteric behavior due to the reported capacity of free Gal to induce ethylene production through stimulating ACS activity. Whether the differences in ethylene and ripening behavior between the two cultivars are also dependent on changes of other hormones is currently under investigation.

## Supplementary data

Supplementary data are available at *JXB* online.

Detailed description of sugar concentration quantifications.

Table S1. Primers used in qRT–PCR.

Table S2. Sugar metabolism-associated genes from RNA-Seq analyses and their corresponding number of edges.

Fig. S1. Fruit ripening patterns and physicochemical properties of Santa Rosa and Sweet Miriam cultivars throughout development and ripening on the tree.

Fig. S2. Schematic diagram of the workflow used to identify key sugar metabolism-associated genes in this study.

Fig. S3. Relative gene expression of sucrose metabolism-associated pathways.

Fig. S4. Relative gene expression of sorbitol metabolism-associated pathways.

Fig. S5. Relative gene expression of minor sugar metabolism-associated pathways.

## Supplementary Material

Supplementary Material

Supplementary Figs1

Supplementary Figs2

## Abbreviations:

CWINV: cell wall invertase
CytINV: cytosolic invertase
DAFB: days after full bloom
DEG: differentially expressed gene;
FPKM: fragments per kilobase per million reads
Fru: fructose
Gal: galactose
α-GAL: α-galactosidase
β-GAL: β-galactosidase
GALK: galactokinase
GO: Gene Ontology
Gol: galactinol
GolS: galactinol synthase
HK: hexokinase
Glu: glucose
G6P: glucose-6-phosphate
INVINH: invertase inhibitor
Ino: myo-inositol
NAD^+^-SDH: NAD^+^-dependent sorbitol dehydrogenase
PCC: Pearson correlation coefficient
Raf: raffinose
RS: raffinose synthase
Sor: sorbitol
SOX: sorbitol oxidase
S6PDH: sorbitol-6-phosphate dehydrogenase
SPS: sucrose phosphate synthase
Suc: sucrose
SuSy: sucrose synthase
TRE: trehalase
Tre: trehalose
UDP-Gal: UDP-galactose
UDP-Glu: UDP-glucose
VINV: vacuolar invertase
WGCNA: weighted gene co-expression network analysis.
