# Supplementary material for: Sugar metabolism reprogramming in a non-climacteric bud mutant of a climacteric plum fruit during development on the tree
Source: J Exp Bot. 2017 Nov 25;68(21-22):5813–28. doi: 10.1093/jxb/erx391 (PMC5854140; doi:10.1093/jxb/erx391)

Sugar metabolism reprograming in a non-climacteric bud mutant of a climacteric plum fruit

Macarena Farcuh[1], Bosheng Li[1], Rosa Rivero[2], Avi Sadka[3], Eduardo Blumwald[1, *]

[1]Dept of Plant Sciences, University of California, Davis, CA 95616; [2]CEBAS, CSIC, Murcia, Spain,[3]Dept of Fruit Tree Sciences, Volcani Research Center, Bet Dagan, Israel.

SUPPLEMENTARY DATA

Sugar concentration quantification

NMR Analyses: The extraction of the metabolites was done in 50 mg of freeze-lyophilized plum fruit and leaf tissues using methanol:chloroform:water (2:2:1) followed by sonication for 1h. After centrifugation at 10,500 xg for 20 min at 4ºC, the upper hydroalcoholic phase was carefully separated and dried under Speed-Vac for 24h. To evaluate the reproducibility of sample preparation, three portions of each sample were used to perform three extractions. For the ^{1}H-NMR quantification, the dry residue of the hydroalcoholic phase was dissolved in 0.75 mL of deuterium oxide phosphate buffer (150 mM, pH 7.0) measured by a glass BNC electrode (Sigma-Aldrich)) containing 3 mM 4,4- Trimethyl-4-silyl propanoic acid (TSP) as an internal standard. All ^{1}H-NMR spectra were recorded at 298 ^{o}K on a Bruker AVIII HD 500 NMR spectrometer (500.13 MHz for ^{1}H) equipped with a 5 mm CPP BBO cryogenic probe (Bruker Biospin, Germany). ^{1}H spectra were referenced to TSP signal (δ = 0.00 ppm), whereas ^{13}C spectra were referenced to CH-^{1} resonance of α-D-glucose (δ = 93.10 ppm). A standard one-dimensional pulse sequence noesypr1d (recycle delay-90°-t1-90°-tm-90°-acquisition) was used to obtain metabolic profiles of plum fruit and leaf extracts with the 90° pulse length of about 11 µs and t1 of 3 µs. Water suppression was achieved with a weak irradiation during the recycle delay (RD = 2 s) and mixing time (100 ms). Sixty-four transients were collected into 32,768 data points for each spectrum with a spectral width of 8 kHz. An exponential window function with line-broadening factor of 0.5 Hz was applied to free induction decays prior to Fourier transformation (FT). For resonance assignment purposes, ^{1}H-^{1}H-correlation spectroscopy (COSY; cosygpqfpr), ^{1}H-^{1}H-total correlation spectroscopy (TOCSY; mlevphpr) and ^{1}H-^{13}C-heteronuclear single quantum coherence (HSQC; hsqcetgp) two-dimensional NMR spectra was acquired for selected samples. In COSY and TOCSY experiments, 48 transients were collected

into 2,048 data points for each 256 increments with the spectral width of 12 ppm for both dimensions. Phase insensitive mode was used with gradient selection for the COSY experiments whereas the well-known MLEV-17 was employed as the spin-lock scheme in the phase sensitive. The TOCSY experiment (time-proportional phase incrementation) had a mixing time of 100 ms. ^{1}H-^{13}C HSQC NMR spectra were recorded using the gradient selected sequences with 200 transients and 2,048 data points for each of the 1,064 increments. The spectral widths were 6,313 Hz for ^{1}H and 26,410 Hz for ^{13}C for HSQC experiments. The data were Fourier transformed into a 4 x 2k matrix with appropriate apodization functions. ^{1}H NMR spectra were manually corrected for phase and baseline distortions using TOPSPIN version 3.2 (Bruker Biospin) and the spectral region of δ=0.5 to 9.5 was uniformly integrated into 3,166 buckets with width of 0.003 ppm (1.8 Hz) using the Chenomx software version 3.8.3 (Bruker Biospin). The region δ=4.67 to 5.15 was discarded to eliminate the eﬀects of imperfect water presaturation. The spectral areas of all buckets were normalized to the weight of extracts employed for measurements. The intensities of 60 selected ^{1}H resonances due hydro-alcoholic metabolites were measured with respect to the intensity of TSP signal used as internal standard with a concentration of 3 mM.

UHPLC-QTOF-MS/MS analyses: The chemical extraction was done in 50 mg of plum fruit and leaf lyophilized tissues using chloroform:methanol:water (1:2:2) followed by sonication of 1h. Samples were centrifuged at 18,000 xg during 5 min at 4ºC. The supernatant was filtered through a 0.2 µm Whatman syringe filter and these extracts were used for the UHPLC-QTOF-MS/MS analysis. Separation of sugars from plum extracts (fruits and leaves) was performed on a Waters Acquity iClass UHPLC (Waters Corporation, Milford, MA) consisting of a vacuum degasser, an auto-sampler and a binary pump. This instrument was equipped with a BEH Amide UHPLC column (2.1 x 100 mm, 1.7 µm) from Waters Corporation. Acidified water (0.1% formic acid, v/v) and Acetonitrile were used as mobile phases A and B, respectively. The gradient was programmed as follows: 0 min, 99% B; 7 min, 30% B; 7.1 min, 99% B; 10 min, 99% B; and finally, the initial conditions were held for 7 min as a re-equilibration step. The flow rate was set at 0.30 mL/min throughout the gradient. The injection volume was 5 µL and the column temperature was maintained at 40 °C. The UHPLC system was coupled to a quadrupole-time-of-flight (maXis Impact HR Q-TOF-MS, Bruker Daltonik GmbH, Bremen, Germany) orthogonal accelerated Q-TOF mass spectrometer, equipped with an electrospray ionization source (ESI).

Parameters for analysis were set using negative and positive ion modes, with spectra acquired over a mass range from 50 to 1200 m/z. The optimum values of the ESI-MS parameters were: capillary voltage, -4.0 and +4.5 kV; drying gas temperature, 200 °C; drying gas flow, 8.0 L/ min; nebulizing gas pressure, 2 bars; collision RF, 150 Vpp; transfer time 72 µs, and pre-pulse storage, 5 µs. The MS data were processed through Data Analysis 4.0 software (Bruker Daltonics, Bremen, Germany) which provided a list of possible elemental formulas by using the Generate Molecular Formula™ editor. The editor uses a CHNO algorithm, which provides standard functionalities, such as maximum/minimum elemental range, and a sophisticated comparison of the theoretical with the measured isotope pattern (mSigma value), for increasing the confidence in the suggested molecular formula. The widely-accepted accuracy for confirmation of elemental compositions has been established as 5 ppm. At some stage in the UHPLC method development, an external apparatus calibration was performed using a KD Scientific syringe pump (Vernon Hills, IL) directly linked to the interface, passing a solution of sodium formate with a flow rate of 180 µL/h. Using this method, an exact calibration curve based on numerous cluster masses each differing by 68 Da (CHO_2Na) was obtained. Due to the compensation of temperature drift in the Q-TOF, this external calibration provided accurate mass values for a complete run without the need for a dual sprayer set up for internal mass calibration.

Table S1. List of primers used in qRT-PCR.

Gene name	*Prunus persica* gene ID	Description	Primer Orientation	Primer Sequence (5'-3')
SPS	ppa000622m.g	Sucrose Phosphate Synthase	Forward	AACACTGCCGGACTTACTTGAC
			Reverse	ACCCTTCAGCAGCCATTTC
CWINV	ppa004218m.g	Cell Wall Invertase	Forward	CAGGACCCTTTGGCTTGATAAG
			Reverse	TCCCTTTAGTACACTGCTTGGC
VINV	ppa002334m.g	Vacuolar Invertase	Forward	CCAGTGACAAGGGATTGGATAC
			Reverse	AAGTCCCGAACGCATAGTAATC
CytINV	ppa002625m.g	Cytosolic Invertase	Forward	GGGACATGAGTGGAAGGTTATC
			Reverse	GCAGCGGTAAACAACCAAAG
INVINH	ppa011699m.g	Invertase Inhibitor	Forward	GCACCTTGTGGTATGGTGAGTATAAG
			Reverse	GAACCTCCCAACAGTGAAGAAG
SuSy	ppa017606m.g	Sucrose Synthase	Forward	AGTATCGGAACTTGGTGAAGAATG
			Reverse	CTTGGTTTGGTCTGTGATTTGG
Tre	ppa003514m.g	Trehalase	Forward	CGTCATCTCTCTGTCACCAATC
			Reverse	ATGAGAATGAAGAGTGGCTATGG
GALK	ppa004568m.g	Galactokinase	Forward	TGGTACACAATCTGGTGGAATG
			Reverse	CGCACATCAGTAGCACGTATAG
β-GAL	ppa002839m.g	Beta-Galactosidase	Forward	TGCAGCTACATTGGGACATTC
			Reverse	CCTCAAACACTACCACCAGATT
α-GAL	ppa007136m.g	Alpha-Galactosidase	Forward	TTGAAGCCAGTCTCGTAACC
			Reverse	AACCATGAGCGTGACATTAGA
S6PDH	ppa009027m.g	Sorbitol-6- Phosphate-Dehydrogenase	Forward	TTTGATTGCGCTGCTGATTAC
			Reverse	CTTGGTGGTGATGAAGAGATC
NAD^{+}-SDH	ppa007458m.g	NAD+-dependent Sorbitol Dehydrogenase	Forward	CCCAGAAGGAGATTGAAGAAGC
			Reverse	ACTTGTAGTAGCTCTCTCAACTTTAC
GolS	ppa008264m.g	Galactinol Synthase	Forward	GGTGAAGAAATGGTGGGACATA
			Reverse	CTTCTCTTGGTCCTGCTCTTG
RS	ppa001841m.g	Raffinose Synthase	Forward	GCTGGGATTTGTGTTGAAGATG
			Reverse	CAGAGGCTGGAGTAACAACAA
HK	ppa004471m.g	Hexokinase	Forward	CCTCCATTGACTCTGGCATAC
			Reverse	CGCCTTTCCATAGCCTCATTC
MON	ppa003026m.g	SAND protein-related trafficking protein	Forward	GGGAACCTTATATTGGCGTAGG
			Reverse	CTGCTGACGAGGACTACTTATTG

Table S2. A list of the sugar metabolism-associated genes from RNA-Seq analyses and their corresponding number of edges. Sugar metabolism-associated genes, obtained from the differentially expressed genes (DEGs) from RNA-Seq analysis (by GO terms and literature data), organized by module names as indicated in Fig. 4 are shown. Genes in bold type correspond to the 28 sugar metabolism-related genes significantly correlated with sugar contents through PCC analysis (Table 2). Number of edges were estimated using as a cutoff a Weighted Gene Co-expression Network Analysis (WGCNA) edge weight ≥0.5, as indicated in "Materials and Methods".

**Module name**	***Prunus persica* gene IDs**	**Description of sugar-metabolism related genes**	**Abbreviation of sugar-metabolism related genes**	**Number of edges**
blue	**ppa002839m.g**	**beta-galactosidase 16**	**β-GAL, βGAL16**	**194**
blue	**ppa004568m.g**	**Mevalonate/ galactokinase family protein**	**GALK**	**188**
blue	ppa001480m.g	beta-galactosidase 8	β-GAL8	88
blue	**ppa002625m.g**	**cytosolic invertase**	**CytINV, At-A/N-InvE**	**67**
blue	ppa004192m.g	Glucose-1-phosphate adenylyltransferase family protein	APL3	65
blue	ppa000820m.g	arabinose kinase	ARA1,ATISA1,ISA1	60
blue	ppa000755m.g	Glycosyl hydrolase family 38 protein	empty	58
blue	ppa006839m.g	Pectin lyase-like superfamily protein	empty	56
blue	ppa006857m.g	Pectin lyase-like superfamily protein	empty	50
blue	ppa001971m.g	cellulose synthase like G3	AtCSLG3,CSLG3	50
blue	ppa003316m.g	Pyruvate kinase family protein	PKP-ALPHA,PKP1	48
blue	ppa008154m.g	UDP-XYL synthase 6	UXS6	45
blue	ppa022137m.g	Galactosyltransferase family protein	empty	45
blue	ppa009245m.g	xyloglucan endotransglucosylase/hydrolase 8	XTH8	45
blue	ppa004653m.g	cellulase 2	ATCEL2,CEL2	44
blue	ppa015545m.g	glucose-6-phosphate dehydrogenase 2	G6PD2	42
blue	**ppa001535m.g**	**sucrose synthase 4**	**SuSy, AtSUS4**	**40**
blue	ppa000827m.g	Glycosyl transferase, family 35	empty	39
blue	ppa006008m.g	pyruvate dehydrogenase E1 alpha	PDH-E1 ALPHA	33
blue	ppa005162m.g	UDP-glucosyl transferase 78D2	UGT78D2	25
blue	ppa002857m.g	Di-glucose binding protein with Leucine-rich repeat domain	empty	25
blue	ppa001276m.g	stachyose synthase	AtSTS,STS	25
blue	ppa002254m.g	Cellulose-synthase-like C5	AtCSLC05,AtCSLC5	24
blue	ppa012287m.g	cell wall / vacuolar inhibitor of fructosidase 1	AtC/VIF1	23
blue	ppa009023m.g	myo-inositol oxygenase 1	MIOX1	22
blue	ppa001573m.g	sucrose synthase 2	AtSUS2,SSA,SUS2	21

blue	**ppa006549m.g**	**Glucose-6-phosphate/phosphate translocator-related**	**APE2,TPT**	**19**
blue	ppa001334m.g	beta-galactosidase 3	β-GAL3	18
blue	ppa006179m.g	alpha-galactosidase 1	AGAL1,AtAGAL1	18
blue	ppa002894m.g	Glycosyl hydrolase family protein	empty	17
blue	ppa011478m.g	Plant invertase inhibitor superfamily	INVINH	16
blue	ppa015127m.g	UDP-glucosyl transferase 88A1	UGT88A1	15
blue	ppa004313m.g	inositol 1,3,4-trisphosphate 5/6-kinase 4	AtITPK4,ITPK4	14
blue	ppa001232m.g	Glycosyl hydrolases family 31 protein	empty	11
blue	**ppa004783m.g**	**O-Glycosyl hydrolases family 17 protein**	**empty**	**11**
blue	**ppa005180m.g**	**UDP-Glycosyltransferase superfamily protein**	**empty**	**10**
blue	ppa008811m.g	UDP-Glycosyltransferase superfamily protein	empty	8
blue	ppb025297m.g	UDP-glucosyl transferase 88A1	UGT88A1	8
blue	ppa005520m.g	UDP-Glycosyltransferase superfamily protein	empty	6
blue	ppa009865m.g	phosphoenolpyruvate carboxylase kinase 1	AtPPCK1,PPCK1	5
blue	ppa006392m.g	Pectin lyase-like superfamily protein	empty	4
blue	ppa001932m.g	tonoplast monosaccharide transporter2	TMT2	2
blue	ppa005761m.g	Pectin lyase-like superfamily protein	empty	2
blue	ppa006411m.g	Glucose-6-phosphate/phosphate translocator-related	ARAPPT,CUE1,PPT	1
darkorange2	**ppa007136m.g**	**alpha-galactosidase 2**	**α-GAL, α-GAL2**	**136**
darkorange2	**ppa008264m.g**	**galactinol synthase 4**	**GolS, AtGolS4**	**111**
darkorange2	ppa005045m.g	UDP-glucose 6-dehydrogenase family protein	empty	110
darkorange2	ppa003984m.g	O-Glycosyl hydrolases family 17 protein	empty	108
darkorange2	ppa006626m.g	Galactosyltransferase family protein	empty	106
darkorange2	ppa006836m.g	sedoheptulose-bisphosphatase	SBPASE	105
darkorange2	ppa004996m.g	Pectin lyase-like superfamily protein	empty	97
darkorange2	ppa005818m.g	Pectin lyase-like superfamily protein	empty	96
darkorange2	ppa009350m.g	xyloglucan endotransglucosylase/hydrolase 9	XTH9	95
darkorange2	ppa008964m.g	Galactose mutarotase-like superfamily protein	empty	93
darkorange2	ppa023537m.g	alpha-L-fucosidase 1	AtFUC1,FUC1	91
darkorange2	ppa014982m.g	Pectin lyase-like superfamily protein	empty	91
darkorange2	ppa002271m.g	beta-galactosidase 3	β-GAL3	89
darkorange2	ppa006961m.g	Glycosyltransferase family 29 (sialyltransferase) family protein	empty	85
darkorange2	ppa025914m.g	Glycosyl hydrolase family protein	empty	82
darkorange2	ppa009090m.g	xyloglucan:xyloglucosyl transferase 33	"XET,XTH33"	82
darkorange2	ppa006239m.g	Glycosyl hydrolase superfamily protein	empty	82

darkorange2	ppa001675m.g	Glycosyl hydrolase family protein	empty	75
darkorange2	ppb008505m.g	Pectin lyase-like superfamily protein	empty	68
darkorange2	ppa002417m.g	rhamnose biosynthesis 1	AtRHM1,RHM1,ROL1	59
darkorange2	ppa004644m.g	glycosyl hydrolase 9B1	AtGH9B1,CEL1,GH9B1	58
darkorange2	ppa001718m.g	beta-xylosidase 1	AtBXL1,BXL1	57
darkorange2	ppa000567m.g	cellulose synthase 6	CESA6,E112,IXR2,PRC1	56
darkorange2	ppa008032m.g	UDP-D-glucose/UDP-D-galactose 4-epimerase 5	UGE5	56
darkorange2	ppa004814m.g	O-Glycosyl hydrolases family 17 protein	empty	52
darkorange2	ppa007618m.g	Glucose-1-phosphate adenylyltransferase family protein	CYT1,EMB101,GMP1,SOZ1,VTC1	48
darkorange2	ppa001098m.g	Glycosyl hydrolases family 31 protein	empty	42
darkorange2	ppa005337m.g	alpha-L-fucosidase 1	AtFUC1,FUC1	35
darkorange2	ppa002424m.g	phosphoenolpyruvate carboxykinase 1	PCK1,PEPCK	29
darkorange2	ppa000636m.g	sucrose phosphate synthase 1F	AtSPS1F,SPS1F	28
darkorange2	ppa003430m.g	O-fucosyltransferase family protein	empty	25
darkorange2	ppa026880m.g	alpha-mannosidase 1	MNS1	22
darkorange2	ppa000557m.g	cellulose synthase 6	CESA6,E112,IXR2,PRC1	21
darkorange2	ppa022545m.g	Nucleotide-diphospho-sugar transferase family protein	empty	20
darkorange2	**ppa006167m.g**	**beta glucosidase 11**	**β-GLU11**	**19**
darkorange2	ppa026425m.g	UDP-Glycosyltransferase superfamily protein	empty	19
darkorange2	ppa012444m.g	Plant invertase inhibitor superfamily	INVINH	18
darkorange2	ppa004126m.g	Galactose oxidase/kelch repeat superfamily protein	empty	18
darkorange2	ppa005688m.g	sugar transporter protein 7	STP7	18
darkorange2	ppa004226m.g	beta glucosidase 13	β-GLU13	18
darkorange2	ppa004921m.g	O-fucosyltransferase family protein	empty	17
darkorange2	ppa003047m.g	Plant invertase inhibitor superfamily	INVINH	14
darkorange2	ppa016442m.g	beta glucosidase 11	β-GLU11	13
darkorange2	ppa005052m.g	UDP-glucosyl transferase 71B6	UGT71B6	12
darkorange2	ppa003417m.g	inositol transporter 4	AtINT4,INT4	12
darkorange2	ppa006056m.g	Galactose oxidase/kelch repeat superfamily protein	empty	11
darkorange2	ppa1027206m.g	O-Glycosyl hydrolases family 17 protein	empty	10
darkorange2	ppa002059m.g	UDP-Glycosyltransferase superfamily protein	empty	10
darkorange2	ppa008326m.g	UDP-Glycosyltransferase superfamily protein	GT72B1,UGT72B1	9
darkorange2	ppa006158m.g	FASCICLIN-like arabinogalactan protein 8	FLA8	8
darkorange2	ppa015738m.g	UDP-Glycosyltransferase superfamily protein	empty	8
darkorange2	ppa009846m.g	phosphoenolpyruvate carboxylase kinase 1	AtPPCK1,PPCK1	7

darkorange2	ppa003622m.g	O-fucosyltransferase family protein	empty	7
darkorange2	ppa004485m.g	ADP-glucose pyrophosphorylase family protein	empty	7
darkorange2	**ppa020483m.g**	**UDP-glucosyl transferase 76E2**	**UGT76E2**	**6**
darkorange2	ppa000102m.g	phosphatidylinositol-4-phosphate 5-kinase family protein	FAB1B	6
darkorange2	ppa004344m.g	sugar transporter 14	AtSTP14,STP14	2
darkorange2	ppa026075m.g	Carbohydrate-binding X8 domain superfamily protein	empty	1
darkred	**ppa003514m.g**	**trehalase 1**	**TRE, AtTRE1**	**58**
darkred	**ppa000622m.g**	**sucrose phosphate synthase**	**SPS, AtSPS**	**34**
darkred	ppa007088m.g	Glycosyl hydrolase superfamily protein	empty	33
darkred	ppa007195m.g	beta glucosidase 41	β-GLU41	32
darkred	ppa006613m.g	Nucleotide-sugar transporter family protein	empty	32
darkred	ppa005334m.g	UDP-Glycosyltransferase superfamily protein	empty	31
darkred	ppa005623m.g	UDP-glucosyl transferase 76E2	UGT76E2	31
darkred	ppa022021m.g	Rhamnogalacturonate lyase family protein	empty	30
darkred	ppa009170m.g	Galactose mutarotase-like superfamily protein	empty	29
darkred	ppa002453m.g	rhamnose biosynthesis 1	AtRHM1,RHM1,ROL1	29
darkred	ppa004981m.g	UDP-glucose 6-dehydrogenase family protein	empty	28
darkred	ppa004430m.g	myo-inositol-1-phosphate synthase 2	AtIPS2,ATMIPS2,MIPS2	28
darkred	ppa004829m.g	UDP-Glycosyltransferase superfamily protein	empty	28
darkred	ppa003690m.g	fructokinase-like 2	FLN2	28
darkred	ppa001281m.g	UDP-glucose pyrophosphorylase 3	UGP3	28
darkred	ppa003185m.g	Mannose-binding lectin superfamily protein	empty	28
darkred	ppa002418m.g	galacturonosyltransferase 1	GAUT1,LGT1	27
darkred	ppa004816m.g	UDP-Glycosyltransferase superfamily protein	empty	27
darkred	ppa004703m.g	Pyruvate kinase family protein	empty	26
darkred	ppa005015m.g	Pectin lyase-like superfamily protein	empty	26
darkred	ppa006746m.g	fructose-bisphosphate aldolase 2	FBA2	25
darkred	ppa005736m.g	mannose-1-phosphate guanylyltransferase (GDP)s	VTC2	25
darkred	ppa002955m.g	UDP-Glycosyltransferase superfamily protein	empty	24
darkred	ppa004909m.g	UDP-glucosyl transferase 85A2	AtUGT85A2,UGT85A2	24
darkred	ppa015619m.g	beta glucosidase 40	β-GLU40	22
darkred	ppa009863m.g	xyloglucan endotransglucosylase/hydrolase 16	XTH16	21
darkred	ppa002446m.g	Deoxyxylulose-5-phosphate synthase	CLA,CLA1,DEF,DXPS2,DXS	21
darkred	ppa003382m.g	Pyruvate kinase family protein	PKP-ALPHA,PKP1	19
darkred	ppa007943m.g	xyloglucan endotransglucosylase/hydrolase 32	XTH32	19

darkred	ppa006911m.g	glucosyl transferase family 8	GATL9,LGT8	18
darkred	ppa017816m.g	beta-glucosidase 47	β-GLU47	18
darkred	ppa003424m.g	O-fucosyltransferase family protein	empty	18
darkred	ppa003986m.g	phosphofructokinase 4	PFK4	17
darkred	ppa001941m.g	cellulose synthase like E1	AtCSLE1,CSLE1	17
darkred	ppa006572m.g	Galactose oxidase/kelch repeat superfamily protein	empty	17
darkred	**ppa008438m.g**	**UDP-galactose transporter 3**	**AtUTR3,UTR3**	**17**
darkred	ppa004989m.g	UDP-glucosyl transferase 85A3	AtUGT85A3,UGT85A3	17
darkred	ppa008275m.g	xyloglucan endotransglucosylase/hydrolase 28	AtXTH28,EXGT-A2,XTH28,XTR2	16
darkred	ppa007744m.g	Aldolase superfamily protein	empty	16
darkred	ppa009472m.g	xyloglucan endotransglucosylase/hydrolase 6	XTH6	15
darkred	**ppa005599m.g**	**Pectin lyase-like superfamily protein**	**empty**	**15**
darkred	ppa009608m.g	xyloglucan endotransglucosylase/hydrolase 16	XTH16	15
darkred	ppa006160m.g	alpha-amylase-like	AMY1,ATAMY1	14
darkred	ppa004655m.g	Glycosyltransferase family 61 protein	empty	14
darkred	ppa002626m.g	O-fucosyltransferase family protein	empty	14
darkred	ppa009792m.g	xyloglucan endotransglucosylase/hydrolase 15	XTH15,XTR7	13
darkred	ppa016365m.g	UDP-Glycosyltransferase superfamily protein	empty	13
darkred	ppa004972m.g	UDP-glucosyl transferase 73B3	UGT73B3	12
darkred	ppa001556m.g	UDP-Glycosyltransferase / trehalose-phosphatase family protein	AtTPS6,TPS6	12
darkred	ppa002372m.g	alpha-L-arabinofuranosidase 1	ARAF,ARAF1,ASD1	11
darkred	ppa007996m.g	Galactose oxidase/kelch repeat superfamily protein	empty	11
darkred	ppa000473m.g	cellulose synthase-like D3	AtCSLD3,CSLD3	10
darkred	ppa004033m.g	sucrose-proton symporter 2	AtSUC2,SUC2,SUT1	10
darkred	ppa003178m.g	Galactose-binding protein	empty	10
darkred	ppa005415m.g	Galactose oxidase/kelch repeat superfamily protein	empty	10
darkred	ppa007794m.g	galacturonosyltransferase-like 3	GATL3	9
darkred	ppa005598m.g	glyceraldehyde-3-phosphate dehydrogenase B subunit	GAPB	9
darkred	ppa024989m.g	UDP-Glycosyltransferase superfamily protein	empty	9
darkred	ppa021513m.g	Glycosyl hydrolase family 38 protein	empty	9
darkred	ppa004150m.g	O-fucosyltransferase family protein	empty	8
darkred	ppa006859m.g	sedoheptulose-bisphosphatase	SBPASE	8
darkred	ppa1027170m.g	Arabinanase/levansucrase/invertase	empty	7
darkred	ppa020981m.g	1-deoxy-D-xylulose 5-phosphate reductoisomerase	DXR,PDE129	6
darkred	ppa005572m.g	UDP-glucosyl transferase 73B3	UGT73B3	6

darkred	ppa006402m.g	alpha-amylase-like 2	AMY2,AtAMY2	6
darkred	ppa014706m.g	Di-glucose binding protein with Kinesin motor domain	empty	6
darkred	ppa005160m.g	O-Glycosyl hydrolases family 17 protein	empty	5
darkred	ppa005856m.g	Galactose oxidase/kelch repeat superfamily protein	empty	5
darkred	ppa004320m.g	O-Glycosyl hydrolases family 17 protein	empty	4
darkred	ppa026228m.g	glycosyl hydrolase 9A1	AtGH9A1,DEC,GH9A1,IRX2,KOR,KOR1,RSW2,TSD1	4
darkred	ppa000493m.g	cellulose synthase-like D3	AtCSLD3,CSLD3,KJK	4
darkred	ppa020358m.g	UDP-Glycosyltransferase superfamily protein	empty	4
darkred	ppa014010m.g	arabinogalactan protein 16	AGP16,AtAGP16	3
darkred	**ppa002559m.g**	**Glycosyl hydrolase family protein**	**empty**	**3**
darkred	ppa004968m.g	UDP-glucosyl transferase 85A2	AtUGT85A2,UGT85A2	3
darkred	ppa016463m.g	UDP-glucosyl transferase 74B1	UGT74B1	2
darkred	ppa001412m.g	beta galactosidase 1	β-GAL1	2
darkred	ppa004471m.g	hexokinase-like 1	AtHKL1,HKL1	2
darkred	ppa019241m.g	NAC transcription factor-like 9	NTL9	2
darkred	ppa024549m.g	cell wall / vacuolar inhibitor of fructosidase 2	ATC/VIF2,C/VIF2	2
darkred	ppa010282m.g	Nucleotide-diphospho-sugar transferases superfamily protein	empty	2
darkred	ppa026557m.g	UDP-Glycosyltransferase superfamily protein	AtUGT85A1,UGT85A1	1
darkred	ppa008448m.g	Nucleotide-sugar transporter family protein	empty	1
darkred	ppa019354m.g	UDP-glucosyl transferase 73B3	UGT73B3	1
darkred	ppa008266m.g	Galactose oxidase/kelch repeat superfamily protein	empty	1
darkred	ppa020191m.g	UDP-Glycosyltransferase superfamily protein	GT72B1,UGT72B1	1
darkred	**ppa002511m.g**	**Cellulose-synthase-like C4**	**AtCSLC04,ATCSLC4,CSLC04,CSLC4**	**1**
darkred	ppa019715m.g	Glycosyltransferase family 61 protein	empty	1
yellow4	**ppa017606m.g**	**sucrose synthase 6**	**SuSy, SUS6**	**26**
yellow4	ppa023079m.g	O-Glycosyl hydrolases family 17 protein	empty	23
yellow4	ppa020393m.g	Glycosyl hydrolase superfamily protein	empty	9
yellow4	ppa001135m.g	sucrose synthase 5	ATSUS5,SUS5	7
yellow4	ppa004437m.g	Plant invertase inhibitor superfamily	INVINH	5
yellow4	ppa004643m.g	O-Glycosyl hydrolases family 17 protein	empty	4
yellow4	ppb017057m.g	polyol/monosaccharide transporter 5	AtPLT5,AtPMT5,PMT5	4
yellow4	ppa021570m.g	Inositol 1,3,4-trisphosphate 5/6-kinase family protein	empty	1
lightpink4	**ppa002334m.g**	**Vacuolar invertase**	**VINV, AtBETAFRUCT4**	**11**
lightpink4	ppa005977m.g	Melibiase family protein	empty	5

lightpink3	**ppa011699m.g**	**Plant invertase inhibitor superfamily**	**INVINH**	**20**
lightpink3	ppa009417m.g	xyloglucan endotransglycosylase 6	XTH23,XTR6	19
lightpink3	ppa004732m.g	O-Glycosyl hydrolases family 17 protein	empty	18
lightpink3	ppa000910m.g	phosphoenolpyruvate carboxylase 1	AtPEPC1,ATPPC1,PEPC1,PPC1	18
lightpink3	ppa007628m.g	Galactose mutarotase-like superfamily protein	empty	18
lightpink3	ppa005855m.g	UDP-Glycosyltransferase superfamily protein	empty	15
lightpink3	ppa005150m.g	6-phosphogluconate dehydrogenase family protein	GPDHC1	14
lightpink3	ppa010506m.g	phosphomannomutase	AtPMM,PMM	14
lightpink3	ppa025195m.g	fructose-bisphosphate aldolase 2	FBA2	7
lightpink3	ppa005641m.g	O-Glycosyl hydrolases family 17 protein	empty	5
lightpink3	ppa004086m.g	phosphofructokinase 5	PFK5	4
lightpink3	**ppa004956m.g**	**UDP-glucosyl transferase 85A2**	**AtUGT85A2,UGT85A2**	**4**
lightpink3	**ppa001889m.g**	**cellulose synthase-like B3**	**ATCSLB03,ATCSLB3,CSLB03**	**3**
lightpink3	ppa011560m.g	Plant invertase inhibitor superfamily	INVINH	2
lightpink3	ppa007237m.g	Nucleotide/sugar transporter family protein	empty	1
lightpink3	ppa004979m.g	UDP-glucosyl transferase 85A2	AtUGT85A2,UGT85A2	1
lightpink3	**ppa005294m.g**	**UDP-glucosyltransferase 74F2**	**AtSAGT1,GT,SAGT1,SGT1,UGT74F2**	**1**
thisthle	ppa019851m.g	xylosyltransferase 1	ATXT1,XT1,XXT1	5
thisthle	ppa009346m.g	xyloglucan endotransglycosylase 6	XTH23,XTR6	1
darkseagreen2	ppa007982m.g	galactinol synthase 1	AtGolS1,GolS1	3
darkseagreen2	ppa001301m.g	trehalose-phosphatase/synthase 9	ATTPS9,TPS9	3
darkseagreen2	ppa002927m.g	O-fucosyltransferase family protein	empty	1
darkseagreen2	ppa015829m.g	pectin methylesterase inhibitor 1	PME1	1
darkseagreen3	ppa006130m.g	Pectin lyase-like superfamily protein	empty	3
darkseagreen3	ppa004719m.g	cellulase 3	ATCEL3,ATGH9B3,CEL3	2
darkseagreen3	**ppa001730m.g**	**Raffinose synthase/ seed imbibition 2**	**RS, AtSIP2,SIP2**	**1**
lightblue4	ppa005846m.g	Mannose-6-phosphate isomerase, type I	MEE31,PMI1	13
lightblue4	ppa006530m.g	Galactosyltransferase family protein	empty	11
lightblue4	ppa008804m.g	myo-inositol oxygenase 4	MIOX4	10
lightblue4	ppa005142m.g	O-Glycosyl hydrolases family 17 protein	empty	7
lightblue4	ppa007079m.g	Glycosyl hydrolase superfamily protein	empty	2
lightblue4	ppa009365m.g	xyloglucan endotransglucosylase/hydrolase 10	XTH10	1

coral1	**ppa001841m.g**	**Raffinose synthase/ seed imbibition 1**	**RS, AtSIP1,SIP1**	**11**
coral1	ppa007685m.g	xyloglucan endotransglucosylase/hydrolase 30	XTH30,XTR4	8
coral1	ppa018204m.g	cellulose synthase like E1	ATCSLE1,CSLE1	2
coral1	**ppa005277m.g**	**UDP-glucosyl transferase 71B6**	**UGT71B6**	**2**
coral1	ppa006453m.g	monogalactosyldiacylglycerol synthase 2	AtMGD2,MGD2	1
coral1	**ppa003718m.g**	**beta glucosidase 15**	**β-GLU15**	**1**
coral1	**ppa007869m.g**	**UDP-glucosyl transferase 71C3**	**UGT71C3**	**1**
lightsteelblue1	ppa005210m.g	O-Glycosyl hydrolases family 17 protein	empty	28
lightsteelblue1	ppa006612m.g	Galactosyltransferase family protein	empty	27
lightsteelblue1	ppa004287m.g	glucosidase 1	GCS1,KNF	23
lightsteelblue1	ppa001363m.g	beta-galactosidase 3	β-GAL3	23
lightsteelblue1	ppa007396m.g	Glycosyl hydrolase family protein with chitinase insertion domain	empty	23
lightsteelblue1	ppa004172m.g	glycosyl hydrolase 9B8	AtGH9B8,GH9B8	22
lightsteelblue1	ppa000209m.g	Pyruvate phosphate dikinase, PEP/pyruvate binding domain	GWD,GWD1,SEX1,SOP,SOP1	21
lightsteelblue1	ppa004534m.g	O-Glycosyl hydrolases family 17 protein	empty	16
lightsteelblue1	ppa020432m.g	O-Glycosyl hydrolases family 17 protein	empty	14
lightsteelblue1	ppa005206m.g	O-Glycosyl hydrolases family 17 protein	empty	13
lightsteelblue1	ppa027011m.g	endoxyloglucan transferase A3	AtXTH27,EXGT-A3,XTH27	11
lightsteelblue1	ppa011318m.g	Plant invertase inhibitor superfamily	INVINH	11
lightsteelblue1	ppa025159m.g	glycosyl hydrolase 9B18	AtGH9B18,GH9B18	10
lightsteelblue1	ppa005375m.g	O-Glycosyl hydrolases family 17 protein	empty	8
lightsteelblue1	ppa024083m.g	UDP-glucosyl transferase 71D1	UGT71D1	8
lightsteelblue1	ppa009437m.g	xyloglucan endotransglucosylase/hydrolase 32	XTH32	5
lightsteelblue1	ppa004881m.g	Pectin lyase-like superfamily protein	empty	4
lightsteelblue1	ppa002370m.g	O-fucosyltransferase family protein	empty	2
lightsteelblue1	ppa005075m.g	UDP-glucosyl transferase 88A1	UGT88A1	1
paleovioltred1	ppa000357m.g	ketose-bisphosphate aldolase class-II family protein	empty	21
paleovioltred1	ppa015713m.g	Xyloglucan endotransglucosylase/hydrolase family protein	TCH4,XTH22	16
paleovioltred1	ppa004523m.g	beta-glucosidase 45	BGLU45	14
paleovioltred1	ppa002571m.g	Phosphofructokinase family protein	empty	13
paleovioltred1	ppa011607m.g	Plant invertase inhibitor superfamily	INVINH	10
paleovioltred1	ppa011831m.g	Plant invertase inhibitor superfamily	INVINH	7
paleovioltred1	ppa006622m.g	Galactosyl transferase GMA12/MNN10 family protein	empty	3
paleovioltred1	ppa005112m.g	UDP-Glycosyltransferase superfamily protein	empty	2
paleovioltred1	ppa004218m.g	cell wall invertase 4	AtcwINV4,cwINV4	1

paleovioltred1	ppa009610m.g	xyloglucan endotransglycosylase 6	XTH23,XTR6	1
paleovioltred1	ppa003518m.g	Phosphofructokinase family protein	empty	1
paleovioltred1	ppa005043m.g	UDP-glucosyl transferase 85A2	AtUGT85A2,UGT85A2	1
chocolate4	ppa002606m.g	Galactosyltransferase family protein	empty	4

Fig. S3. Relative gene expression levels of Sucrose metabolism-associated pathways. Relative gene expression levels of sucrose metabolism-associated pathways in (A) fruits and (B) leaves of Santa Rosa (SR; black bars) and Sweet Miriam (SM; white bars) throughout development (S2 (pit hardening)) and ripening (S3/S4 (mature), S4-I (commercial harvest), S4-II (fully-ripe)) stages on the tree. Values were calculated and normalized using the SAND protein-related trafficking protein (*MON*) as reference gene and are presented as means ± SE (n=6). The data were analyzed using two-way ANOVA followed by Tukey's test. Different letters indicate significant differences ($p<0.05$). Sucrose phosphate synthase (*SPS*); Sucrose synthase (*SuSy*); cell wall invertase (*CWINV*); vacuolar invertase (*VINV*); cytosolic invertase (*CytINV*); Invertase inhibitor (*INVINH*).

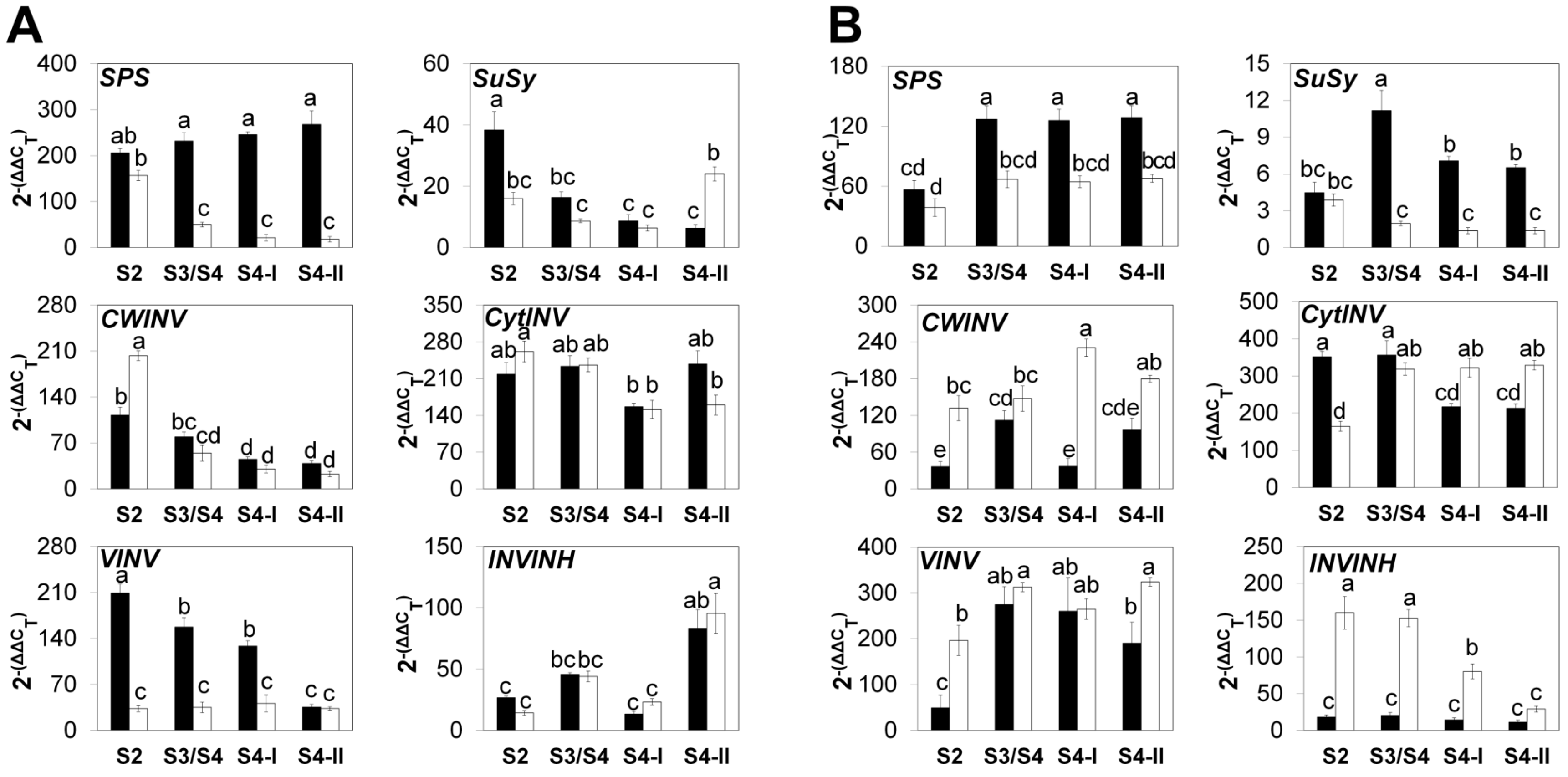

Fig. S4. Relative gene expression levels of Sorbitol metabolism-associated pathways. Relative gene expression levels of sorbitol metabolism-associated pathways in (A) fruits and (B) leaves of Santa Rosa (SR; black bars) and Sweet Miriam (SM; white bars) throughout development (S2 (pit hardening)) and ripening (S3/S4 (mature), S4-I (commercial harvest), S4-II (fully-ripe)) stages on the tree. Values were calculated and normalized using the SAND protein-related trafficking protein (*MON*) as reference gene and are presented as means ± SE (n=6). The data were analyzed using two-way ANOVA followed by Tukey's test. Different letters indicate significant differences (p<0.05). Sorbitol-6-phosphate dehydrogenase (*S6PDH*); NAD$^+$-dependent sorbitol dehydrogenase (*NAD$^+$-SDH*); hexokinase (*HK*).

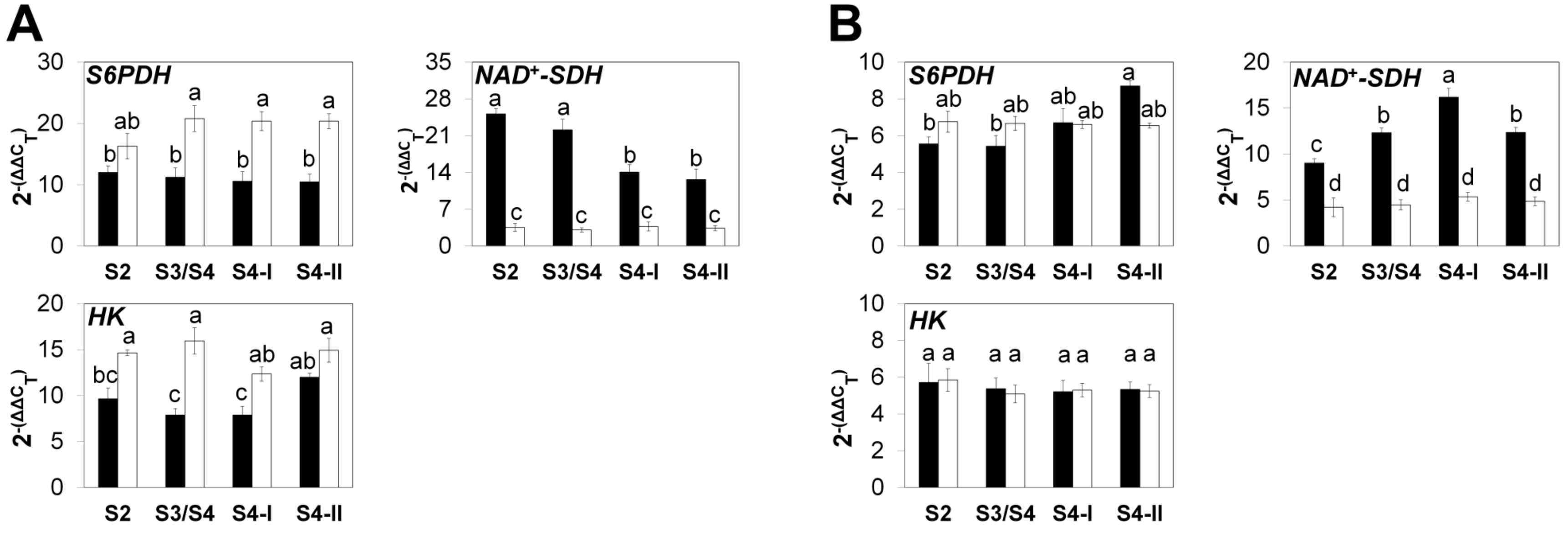

Fig. S5. Relative gene expression levels of minor sugar metabolism-associated pathways. Relative gene expression levels of galactose, galactinol, raffinose and trehalose metabolism-associated pathways in (A) fruits and (B) leaves of Santa Rosa (SR; black bars) and Sweet Miriam (SM; white bars) throughout development (S2 (pit hardening)) and ripening (S3/S4 (mature), S4-I (commercial harvest), S4-II (fully-ripe)) stages on the tree. Values were calculated and normalized using the SAND protein-related trafficking protein (*MON*) as reference gene and are presented as means ± SE (n=6). The data were analyzed using two-way ANOVA followed by Tukey's test. Different letters indicate significant differences ($p<0.05$). Alpha-Galactosidase (α-GAL); Beta-Galactosidase (*β-GAL*); Galatokinase (*GALK*); Galactinol synthase (*GolS*); Raffinose synthase (*RS*); Trehalase (*TRE*).

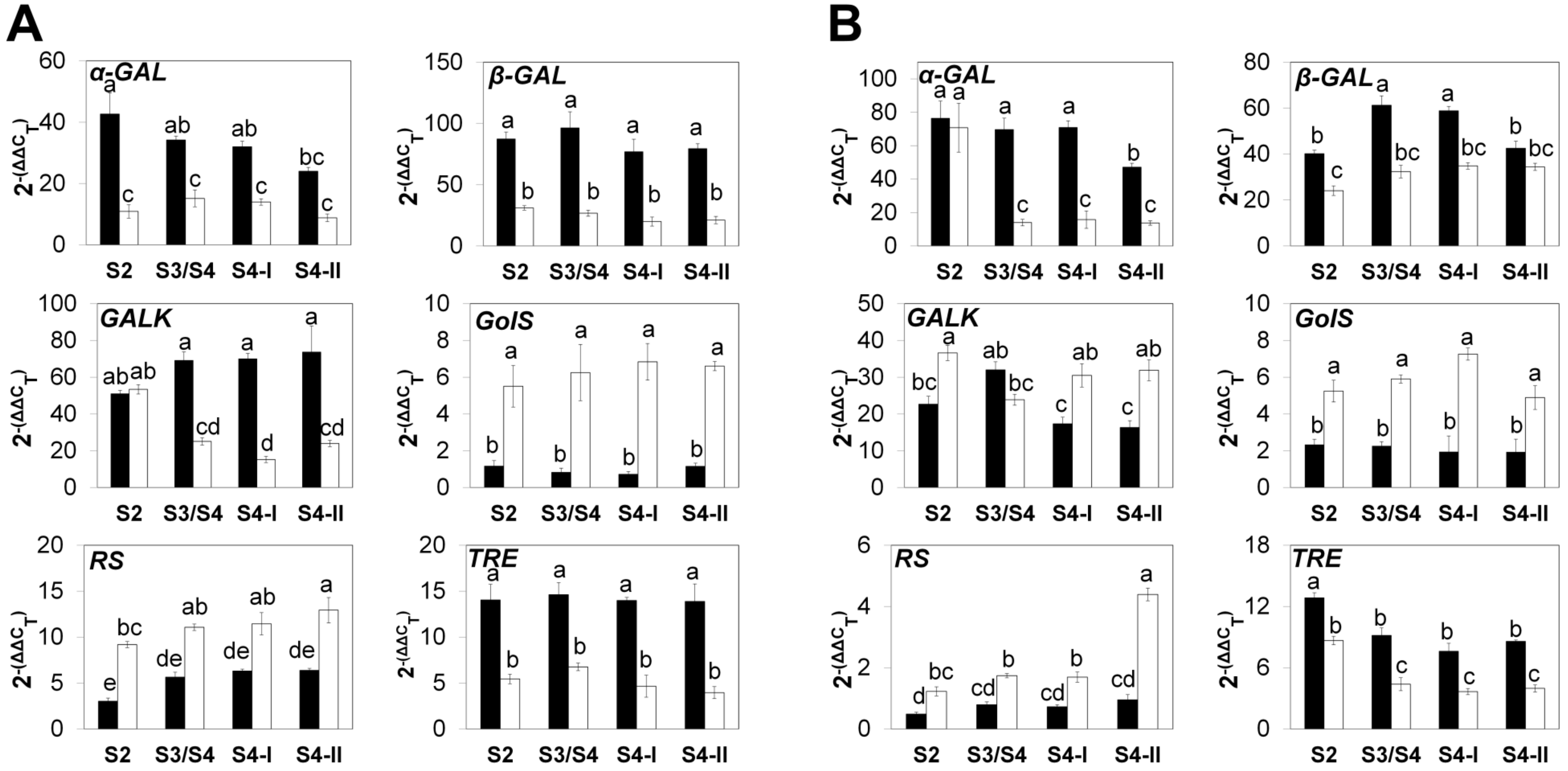

Supplement: Supplementary Material [file erx391_suppl_supplementary_tables_s1_s2_figures_s3_s5.pdf]
