# Supplementary figures and images for: Sugar metabolism reprogramming in a non-climacteric bud mutant of a climacteric plum fruit during development on the tree
Source: J Exp Bot. 2017 Nov 25;68(21-22):5813–28. doi: 10.1093/jxb/erx391 (PMC5854140; doi:10.1093/jxb/erx391)

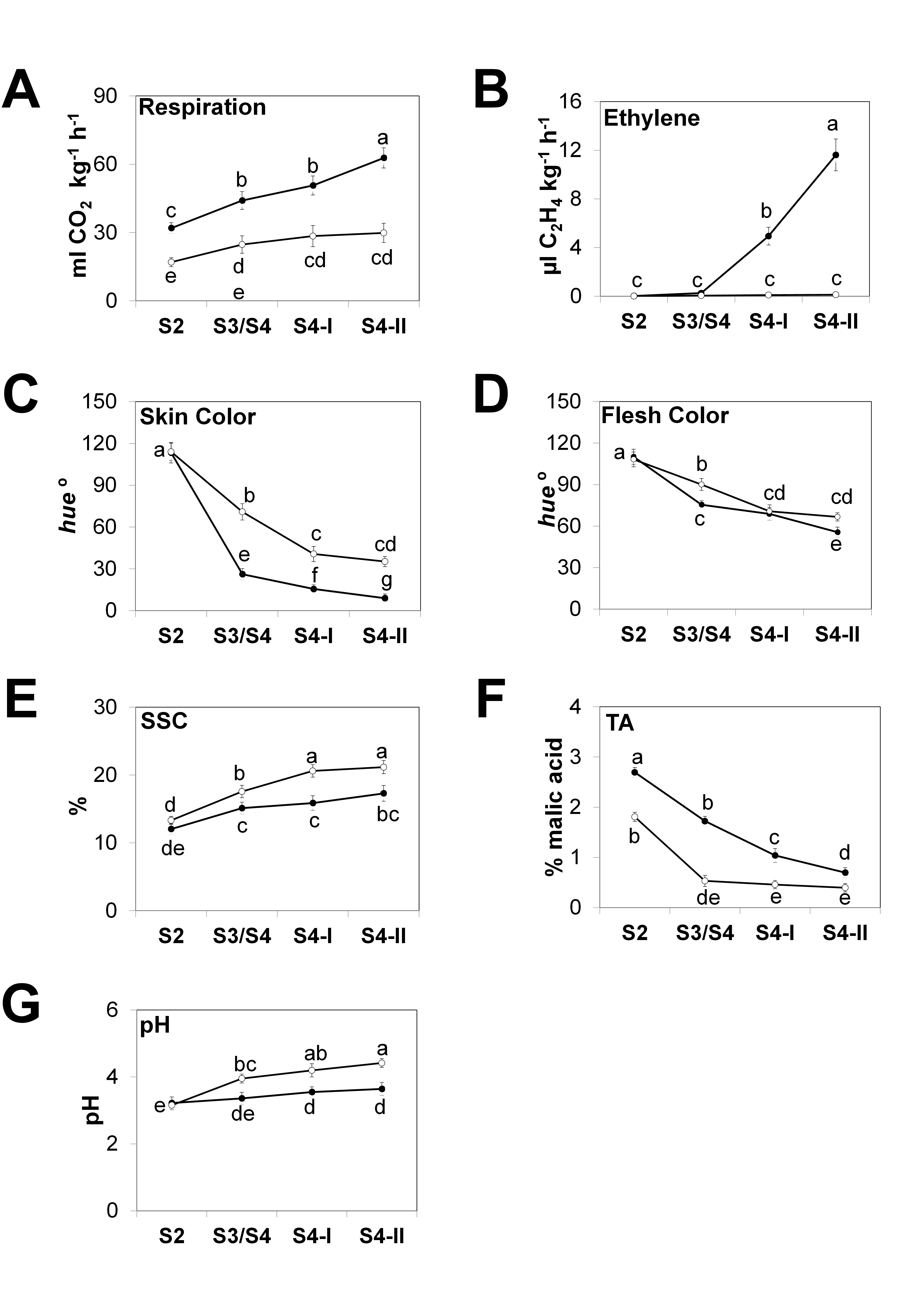

Supplement: Supplementary Figs1 [file erx391_suppl_supplementary_figs1.png]

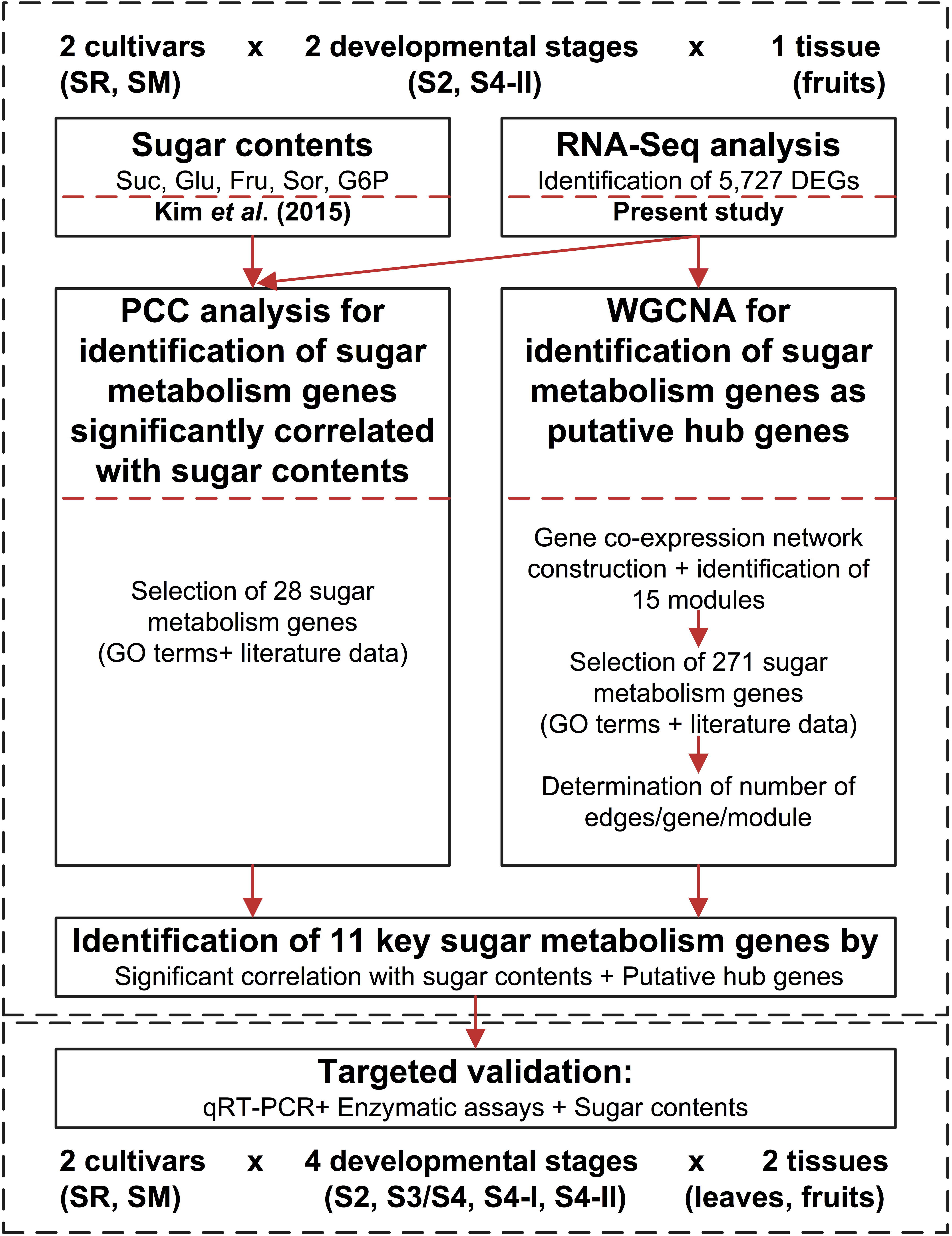

Supplement: Supplementary Figs2 [file erx391_suppl_supplementary_figs2.png]
